# Genomic profiling of ESBL/AmpC-producing *Escherichia coli* from backyard poultry: resistome, virulome, plasmidome, and CRISPR-Cas insights

**DOI:** 10.3389/fmicb.2026.1836952

**Published:** 2026-05-29

**Authors:** Pavan Kalyan Nagaraja, Susweta Das Mitra, Devi Murugesan, Praveen Kumar Attiganahalli Muninarayanaswamy, Sujatha Geddam, Nimita Venugopal, Rituparna Tewari, Aishwarya Singanayakanahalli Ramamurthy, Somy Skariah, Shivasharanappa Nayakvadi, Bibek Ranjan Shome, Rajeswari Shome

**Affiliations:** 1 ICAR-National Institute of Veterinary Epidemiology and Disease Informatics, Bengaluru, India; 2 Department of Biotechnology, School of Basic & Applied Sciences, Dayananda Sagar University, Bengaluru, India; 3 Department of Microbiology, M.S. Ramaiah College of Arts, Science, and Commerce, Bengaluru, India

**Keywords:** AMR, ESBL/AmpC β-lactamases, *Escherichia coli*, India, poultry, WGS

## Abstract

**Introduction:**

Antimicrobial resistance (AMR) is a major One Health concern driven by many factors including unregulated animal farming. This study aimed to perform a comprehensive phenotypic and genotypic characterization of *Escherichia coli* isolated from backyard poultry reared in households in rural setup in southern India.

**Methods:**

A total of 48 cloacal samples were collected from poultry birds across 12 epidemiological units using a 30-cluster sampling strategy. *E. coli* isolation was performed using standard microbiological methods and confirmed by species-specific multiplex PCR. Of the 48 samples, 45 yielded confirmed *E. coli* isolates, among which 18 ESBL- and/or AmpC-positive isolates were selected for whole genome sequencing (WGS). Genomic analyses included detection of antimicrobial resistance genes, virulence-associated genes, mobile genetic elements, plasmid replicons, biofilm-associated genes, multilocus sequence typing, serotyping, CH typing, Clermont phylogrouping, CRISPR-Cas profiling, and SNP-based phylogenetic analysis.

**Results:**

A total of 48 cloacal samples yielded 45 confirmed isolates of which 18 detected with ESBL genes including *bla*_SHV_ (28.8%), *bla*_TEM_ (26.6%), and *bla_CTX-M_* (20%) along with plasmid-mediated *AmpC* genes (11.11%). Resistome profiling revealed diverse ARGs such as *bla*_*CTX-M-15*,_*bla*_DHA-1_, *qnrS/B, tet(A), sul1/2/3, dfrA* var*iants,* and *mphA/B,* in addition to intrinsic efflux systems (*acr, emr, mdt, mar*). Virulome analysis showed conserved genes associated with adhesion *(fim, csg, ecp),* iron acquisition *(ent, fep, fes, ybt),* and stress response *(gad, hlyE, ompT, iss).* CRISPR-Cas analysis revealed a high prevalence of Type I-E arrays (83.3%) coexisting with multiple plasmids. MLST and CH typing revealed high genetic diversity across 12 sequence types, including ST10, ST48, ST3107, and ST226. SNP-based phylogeny placed isolates mainly within commensal phylogroups A and B1, with relatedness to global strains. All sequenced isolates were multidrug-resistant, with significant *β*-lactam resistance enrichment (Z = 3.46, *p* < 0.001). ARG distribution differed significantly among phylogroups (*p* = 0.036), and Simpson’s diversity index (D = 0.94) indicated marked clonal heterogeneity. Strong plasmid–ARG associations (Cramér’s V > 0.5) suggested plasmid-mediated resistome structuring.

**Discussion:**

Backyard poultry-derived *E. coli* showed multidrug resistance, genetic diversity, and virulence-associated traits, highlighting backyard poultry as a potential reservoir for AMR dissemination at the human–animal–environment interface.

## 1 Introduction

Maintaining the health of livestock and poultry is important to support food security and economic stability of nations as it supports the welfare of people globally (Kappes et al., 2023; Robinson et al., 2016). Poultry production is one of the fastest-growing animal agriculture sectors worldwide and is made possible due to low input costs, high-value output products and the universal acceptance of poultry products by consumers. As of 2024, the commercial poultry sector accounts for approximately 98% of global poultry meat production and 92% of global egg production (FAO, 2024). Chickens dominate commercial production, and the projections for the 2025–2034 periods indicate that this intensification will continue particularly in developing economies (Organisation for Economic Co-operation and Development-Food and Agriculture Organization of the United Nations, 2025).

The global expansion of antimicrobial resistance (AMR) has emerged as a critical One Health challenge particularly in food animal production systems. Recent estimates indicate that bacterial AMR was directly responsible for approximately 1.27 million deaths globally in 2019 with a substantial burden linked to food-producing animal systems (Murray et al., 2022). Poultry production systems due to their rapid intensification, high stocking densities, and frequent antimicrobial exposure are recognized as important reservoirs for AMR bacteria and resistance genes (Nhung et al., 2017; Roth et al., 2019). The use of antimicrobials for therapeutic, prophylactic, and growth-promoting purposes exerts strong selection pressure on gut microbiota facilitating the emergence and persistence of resistant bacterial populations. However, in smallholder backyard poultry systems, antimicrobial usage patterns may be variable and not always well documented, and resistance may also arise through environmental exposure, contact with other livestock, or introduction of resistant strains from external sources.

Among these, *Escherichia coli*, a ubiquitous commensal bacterium of the gastrointestinal tract serves as a key reservoir of antimicrobial resistance genes (ARGs) and plays a central role in their dissemination across animal, environmental, and human interfaces (Murray et al., 2022). Its genomic plasticity and large accessory genome enable rapid adaptation under selective pressures allowing transition from a benign commensal organism to an opportunistic pathogen.

One of the most abundant members of the gut microbiota is *E. coli* which has many important functions such as metabolism of nutrients, exclusion of pathogenic bacteria and support of the maturation of the immune system. However, due to the exceptional genomic plasticity of the *E. coli* genome and the large *E. coli* pangenome, the organism is capable of changing rapidly from being a benign commensal bacterium to an opportunistic pathogen in response to gut dysbiosis, inflammation or environmental stresses (Braz et al., 2020; Ramos et al., 2020). Its ability to acquire and disseminate virulence determinants such as adhesins, toxins, siderophores, invasins and biofilm operons and resistance genes including ESBLs (*bla*_CTX-M_, *bla*_TEM_ and *bla*_SHV_), AmpC*, qnr, tet, sul* and *aac*-family genes makes it a highly adaptable and clinically significant organism (Ramos et al., 2020; Hussain et al., 2019; Das et al., 2022). Avian pathogenic *E. coli* (APEC) serves as a key reservoir for these resistance and virulence traits with clear zoonotic potential (Poirel et al., 2018; Mellata, 2013).

The AMR threat is further amplified by mobile genetic elements (MGEs) such as plasmids, insertion sequences and transposons which often carry clusters of resistance and virulence genes (Johansson et al., 2021; Jarocki et al., 2020; Ramos et al., 2020). MGEs are known to facilitate the movement of resistance determinants across bacterial populations, spreading of MDR phenotypes within and between farms enabling resistant strains to disseminate into soil, water and surrounding environments via fecal shedding. This environmental dissemination allows ARB to persist and spread to humans, animals and wildlife (Wee et al., 2020; Hickman et al., 2021). Reports also highlight increasing transfer of MDR from *E. coli, Klebsiella pneumoniae* and *Staphylococcus aureus* between animals and humans in regions with high livestock density and inadequate biosecurity measures (Braz et al., 2020; Jarocki et al., 2020; Ludden et al., 2018; Matsukawa et al., 2019).

Despite the global recognition of AMR to poultry birds, genomic characterization of poultry-associated *E. coli* remains limited within smallholder backyard poultry farming systems that dominate much of rural poultry production in India (Braga et al., 2016; Ghatak et al., 2021; Ahmad et al., 2023). Traditional phenotypic antimicrobial susceptibility testing provides essential information on resistance patterns. However, deeper genomic features of resistome, virulome, serotype, MLST profile, phylogroup, biofilm genes, CRISPR-Cas systems and MGE composition often remain undetected. Analysis of CRISPR-Cas systems in *E. coli* is specifically important in AMR studies because it reveals how bacterial adaptive immunity may limit or when attenuated, permit the acquisition and persistence of plasmid-borne resistance genes influencing the spread of MDR.

Whole genome sequencing (WGS) combined with core genome single nucleotide polymorphism (SNP) phylogenomics enables high-resolution analysis of bacterial evolution, clonal relatedness and global transmission pathways offering unparalleled insights into AMR dynamics (Dierikx et al., 2020; Tantoso et al., 2022; Zhao et al., 2024). This integrated genomic approach is essential for understanding the emergence and spread of high-risk clones and for informing targeted interventions under a One Health framework (Aslam et al., 2021; Lehtinen et al., 2019).

This study addresses these critical knowledge gaps by conducting an integrated phenotypic, molecular and genomic investigation of *E. coli* isolates to derive insights into the AMR mechanisms, virulence potential and phylogenetic placement of Indian poultry-derived *E. coli.* The findings contribute to national AMR surveillance efforts and offer foundational evidence to support targeted mitigation strategies even at the backyard poultry production systems.

## 2 Materials and methods

### 2.1 Study design and sampling plan

A cross-sectional study was conducted to isolate *E. coli* from poultry birds reared at backyard farming systems. A clustered sampling design was adopted in which Chikkaballapur district of Karnataka, India, was chosen as a primary sampling unit followed by four geographically separated blocks (Chikkaballapur, Gauribidanur, Sidlaghatta and Chintamani) at proximity of approximately 30 km apart. In each of the block, three epidemiological units (villages) were selected that were ideally separated by 5-10 km representing the third sampling stratum (Thrusfield, 2018). In each village, 5 households (HH) of farmers that rear backyard poultry birds were selected based on farmer willingness to participate. In total, 48 cloacal samples collected from poultry birds across 12 epiunits. This structured design allowed us to gather robust and representative samples in unorganized poultry systems and accommodate for the impact of clustering bias (Kumar et al., 2024; ICAR-NIVEDI Annual Report, 2019). Together with samples from poultry birds, milk from cattle and buffaloes, rectal swabs from sheep and goats and pigs were also collected from livestock. A total of 48 cloacal samples were collected from poultry birds across 12 epidemiological units (epiunits). Of these, 45 samples yielded confirmed *E. coli* isolates based on biochemical and molecular identification. Phenotypic screening identified 18 isolates exhibiting ESBL and/or AmpC characteristics, all of which were further confirmed by PCR for *β*-lactamase genes. These 18 isolates were subsequently selected for WGS and downstream genomic analyses.

### 2.2 Sample and metadata collection

Metadata corresponding to each sampling location were documented using a structured questionnaire, including village name, farmer details, sampling date and GPS coordinates (latitude/longitude). Additional epidemiological information such as flock size, rearing and feeding practices, hygiene measures, vaccination status and recent disease history was also recorded. Although metadata on management practices were collected, detailed quantitative information on antimicrobial usage was not consistently available across all households. The collected metadata were used to provide epidemiological context for the sampled populations; however, detailed spatial association analysis was not performed in the present study. All datasets were compiled in Microsoft Excel 2010 and geographic mapping of sampling sites was performed using QGIS v3.14 (QGIS Development Team, 2020). The 48 cloacal swab samples were collected between January and April 2019 using sterile cotton swabs directly from freshly voided droppings or cloacal sites and transported on cold-chain conditions to the laboratory following standard ICAR guidelines for biosample handling (ICAR-NIVEDI Annual Report, 2019).

### 2.3 Phenotypic and genotypic identification of *Escherichia coli*

Cloacal swabs were enriched in MacConkey Lactose Broth (MLB) at 37 °C for 18–24 h and streaked onto MacConkey Lactose Agar (MLA) and incubated as mentioned above. Lactose-fermenting colonies were further purified by sub-culturing twice on Brain Heart Infusion Agar (BHA). Phenotypic identification involved Gram staining, IMViC reactions, lactose fermentation and oxidase testing (Jagana et al., 2023). For molecular confirmation, genomic DNA was extracted as per the manufacturer’s instructions (California, United States) and DNA concentration and purity were assessed using a NanoDrop 2000 spectrophotometer (Thermo Scientific, Waltham, United States) and verified on a 1.5% agarose gel. Definitive confirmation of *E. coli* was achieved through multiplex PCR (mPCR) targeting multiple species-specific loci including *uidA* (*β*-D-glucuronidase), *lacZ* (β-D-galactosidase), *lacY* (lactose permease), *cydA* (cytochrome bd oxidase complex), and *phoA* (bacterial alkaline phosphatase). PCR-amplified products were run on 1.5% agarose gels stained with ethidium bromide and visualized using the ChemiDoc MP Imaging System (Bio-Rad, United States) (Mitra et al., 2024).

### 2.4 *In vitro* antimicrobial susceptibility testing (AST)

AST of *E. coli* isolates was performed using the Kirby–Bauer disk diffusion method on Mueller–Hinton Agar (MHA) plates (Himedia, Mumbai, India) following the Clinical and Laboratory Standards Institute (CLSI) (2021) guidelines. A total of seventeen antibiotics representing twelve antimicrobial classes were evaluated in accordance with CLSI guidelines [aminoglycosides (amikacin, 30 μg), β-lactam/β-lactamase inhibitor combination (amoxicillin–clavulanic acid, 20/10 μg), aminopenicillins (ampicillin, 10 μg), cephalosporins third-generation (ceftiofur, 30 μg; cefpodoxime, 10 μg; ceftazidime, 30 μg; cefotaxime, 30 μg; ceftriaxone, 30 μg), cephamycin (cefoxitin, 30 μg), monobactams (aztreonam, 30 μg), carbapenems (imipenem, 10 μg), quinolones (nalidixic acid, 30 μg), fluoroquinolones (enrofloxacin, 5 μg), tetracyclines (tetracycline, 30 μg), folate pathway inhibitors (trimethoprim–sulfamethoxazole, 1.25/23.75 μg), phenicols (chloramphenicol, 30 μg) and polymyxins (colistin, 10 μg)]. After incubation at 37 °C for 18–24 h, the zone of inhibition in diameters were measured and interpreted as susceptible, intermediate or resistant according to CLSI breakpoints. Antibiotic susceptibility results were managed and analyzed using WHONET v5.6. Multidrug resistance (MDR) was defined as non-susceptibility (resistant + intermediate) to least at one agent in three or more antimicrobial classes (Magiorakos et al., 2012). Similar methodologies and MDR definitions have been applied in Indian swine and poultry studies supporting standardized comparisons of resistance patterns (Jagana et al., 2023).

### 2.5 Phenotypic and genotypic identification of ESBL and *AmpC* genes

Phenotypic detection of ESBL and AmpC production was performed using the double disc diffusion assay following CLSI guidelines. Overnight growth of *E. coli* cultures were adjusted to 0.5 McFarland turbidity and inoculated onto Mueller–Hinton agar plates (MHA). Plates were incubated at 37 °C for 18–24 h and interpreted as per CLSI breakpoints. Discs of cefpodoxime, ceftazidime, aztreonam, cefotaxime, and ceftriaxone were applied with ceftazidime and cefotaxime placed 20 mm from amoxicillin–clavulanic acid for ESBL confirmation. Enhancement of the inhibition zone towards clavulanate was interpreted as ESBL production. Isolates showing resistance to multiple cephalosporins without clavulanate synergy were considered presumptive AmpC producers.

Genomic DNA was extracted from purified *E. coli* cultures as mentioned earlier and PCR assays were performed to detect genes encoding ESBL enzymes (*bla*_CTX-M,_ *bla*_TEM_, and *bla*_SHV_) as well as the *AmpC* gene encoding *AmpC β*-lactamase following the standardized protocol described (Mitra et al., 2024; Dallenne et al., 2010). *E. coli* ATCC 25922 was used as a quality control strain for AST. For PCR assays previously confirmed ESBL and *AmpC* genes positive isolates were used as positive controls.

### 2.6 Whole genome sequence analyses

Genomic DNA from all *E. coli* isolates was extracted and DNA concentration and purity were assessed by spectrophotometer before WGS analysis. Genomic libraries were prepared using the Nextera XT DNA Library Preparation Kit (Illumina, United States) according to the manufacturer’s protocol. WGS was performed on Illumina HiSeq 2,500 and MiSeq platforms using paired-end chemistry (2 × 250 bp and 2 × 300 bp). Raw reads were subjected to quality assessment using FastQC v0.11.9^1^ and subsequently processed to remove adapters, low-quality bases and ambiguous reads using fastp v0.22.0^2^ and Trimmomatic v0.39^3^. Sequencing depth across isolates was sufficient for reliable genome reconstruction with average coverage exceeding 30×. High-quality filtered reads were assembled *de novo* using Shovill v1.0.9^4^ which utilizes SPAdes v3.13.0^5^ as the underlying assembler (Wick et al., 2017). Contigs shorter than 500 bp were removed and genome scaffolding was performed using Medusa v1.6.^6^ Assembly quality was evaluated using standard metrics including total genome size, number of contigs, N50 values, and GC content. Average Nucleotide Identity (ANI) was calculated using FastANI^7^ to assess similarity against the *E. coli* K-12 MG1655 reference genome (NC_000913). Genome annotation was performed using the NCBI Prokaryotic Genome Annotation Pipeline (PGAP)^8^ and additionally with Prokka v1.14.6^9^ (Seemann, 2014). The ARGs were identified using ResFinder v2.1^10^ (Bortolaia et al., 2020; Zankari et al., 2012) and validated using the Resistance Gene Identifier (RGI) from the Comprehensive Antibiotic Resistance Database (CARD)^11^ (Alcock et al., 2023). Virulence-associated genes were detected using VirulenceFinder v2.0^12^ along with manual curation based on previously reported *E. coli* virulence genes (Maluta et al., 2017; Ouellette et al., 2022; Renzhammer et al., 2020). Plasmid replicons were determined using PlasmidFinder.^13^ The MGEs including insertion sequences, transposons and associated ARG/VF linkages were identified using MobileElementFinder v1.0.2^14^ (Johansson et al., 2021). CRISPR-Cas arrays were identified using CRISPRFinder which were used to detect direct repeats, spacer sequences and associated Cas genes.^15^ Evidence levels, CRISPR completeness and subtype classification were curated for each isolate. Spacer–target matching and anti-CRISPR gene screening were not performed in the present study. MLST was performed using the MLST v2.0 server^16^ (Larsen et al., 2012), O: H serotypes were inferred using SerotypeFinder v2.0^17^ and phylogroups were assigned using ClermontTyping^18^ (Clermont et al., 2013). Allelic types of *fimH* and *fumC* were identified using CH Typer^19^ (Roer et al., 2018). Biofilm-associated genes including those related to curli fimbriae, adhesins and outer membrane proteins were identified using curated reference gene sets from previously published studies and specialized biofilm gene repositories (Das et al., 2022; Klein et al., 2018; Sharma et al., 2016; Yan et al., 2020; Schaufler et al., 2019; Pourciau et al., 2020). Heatmaps for gene distribution analyses were generated using R v3.3.2.^20^ Phylogenetic trees were constructed and visualized using IQTree v1.4.2^21^ and iTOL v4^22^ (Letunic and Bork, 2021).

### 2.7 Principal component analysis (PCA)

Multivariate integration of phenotypic resistance (ABST), AMR genes (ARGs), and virulence/biofilm determinants was performed using the Scikit-learn library in python. Resistance phenotypes were numerically encoded (R = 2; I = 1; S = 0), and genotypic presence/absence data were treated as binary. All variables underwent Z-score transformation to standardize scale-disparate datasets before dimensionality reduction via PCA. This approach has been previously validated for high-accuracy prediction of AMR patterns in *E. coli* (Tyson et al., 2015). To define the central distribution of the *E. coli* populations and identify significant drivers of divergence, we calculated 95% confidence ellipses and generated correlation biplots.

### 2.8 Comparative phylogenomics and SNP-based core genome analysis

SNP-based core genome alignment of the *E. coli* isolates generated in this study was performed using Roary v3.12.0 (Page et al., 2015) to define the core genome followed by construction of a maximum-likelihood phylogenetic tree using IQ-TREE (Nguyen et al., 2015). To place the study isolates in a global evolutionary context, a comparative phylogenomic dataset was assembled using publicly available *E. coli* genomes retrieved from NCBI GenBank. The reference dataset comprised 105 genomes from multiple geographic regions (China-48, India-26, Brazil-17, Cuba-13, Ghana-9, Ecuador-5, Thailand-3, and Bangladesh-2) representing Asia, Africa, North America, and South America. Genomes were selected from poultry-associated cloacal, fecal, and cecal sources to ensure ecological comparability. Associated metadata, including GenBank accession numbers, country of origin, geographic grouping, phylogroup assignment, and isolation source were curated and summarized in Supplementary Table S1. The resulting phylogenetic tree was visualized using Interactive Tree of Life (iTOL v5) enabling high-resolution comparison of the study isolates with globally circulating *E. coli* lineages (Letunic and Bork, 2021). Pairwise SNP distance analysis was not performed in the present study; therefore, the phylogenetic relationships inferred are intended to reflect overall genetic relatedness and evolutionary context rather than direct inference of recent transmission events. Associations between AMR, VF, plasmid replicons, and MGEs were evaluated at the isolate level; contig-level co-localization or physical linkage of these features was not systematically resolved.

### 2.9 Statistical analysis

Statistical analyses were performed to evaluate correlations among ARGs, VFs, plasmid replicons, and MGEs, their distributions across phylogroups and STs. Concordance between ARG classes and plasmid replicon types was quantified using Cramér’s V with values >0.5 interpreted as moderate to strong associations. Heatmaps and hierarchical clustering were generated in R v4.3.1 using the pheatmap and complex heatmap packages. Pairwise comparisons of gene prevalence across phylogenetic clusters were performed using Fisher’s exact test or Pearson’s chi-square test as appropriate with a significance threshold of *α* = 0.05. Genetic heterogeneity among isolates based on MLST and serotype profiles was assessed using Simpson’s diversity index (Hunter and Gaston, 1988). Differences in ARGs burden across phylogroups were evaluated using one-way analysis of variance (ANOVA) followed by Tukey’s HSD *post hoc* test (*p* < 0.05). Given the unequal sample sizes across phylogroups, these results were interpreted cautiously and considered exploratory. Biofilm-associated gene counts that deviated from normality were analyzed using the Kruskal–Wallis test to accommodate non-parametric distributions. To evaluate whether the observed prevalence of ESBL/AmpC-producing isolates significantly exceeded expected levels, a one-sample Z-test was performed. The baseline prevalence (20%) was derived from previously reported lower-bound estimates of ESBL prevalence in poultry-associated *E. coli* (Ghatak et al., 2021). This value was selected as a conservative reference point for comparison with the observed prevalence in the present study.

## 3 Results

### 3.1 Phenotypic and genotypic confirmation of *Escherichia coli*

All together 48 cloacal samples collected from poultry across 12 epiunits yielding characteristic lactose fermenting 45 pink colonies on MLA media were shortlisted for purification and biochemical profiling. These 45 colonies showed oxidase negative, catalase positive and IMViC reaction pattern of ++−− and amplified at least two-three species-specific markers by mPCR for the targets such as *lacY, lacZ, cydA, uidA* and *phoA*. Among the markers *uidA* and *phoA* were consistently detected across isolates and *E. coli* ATCC 25922 strain served the positive control ensuring assay reliability and validating the amplification patterns (Supplementary Figure 1A).

### 3.2 Phenotypic and genotypic confirmation of ESBL and *AmpC* genes

Double disc diffusion *in vitro* assay revealed a high prevalence of *β*-lactam resistance among the *E. coli* isolates. Out of 45 isolates, 21 (46.6%) were confirmed as ESBL producers, four (8.8%) exhibited AmpC-only phenotype, and eight (17.7%) showed a combined ESBL and AmpC phenotype. Most ESBL-positive isolates displayed resistance to multiple third-generation cephalosporins including cefpodoxime, ceftazidime, cefotaxime, ceftriaxone, and aztreonam. Isolates co-producing ESBL and AmpC demonstrated broader resistance profiles often showing complete resistance to all tested *β*-lactams. A few isolates exhibited intermediate or sensitive responses to selected agents indicating heterogeneity in resistance pattern.

**Figure 1:**
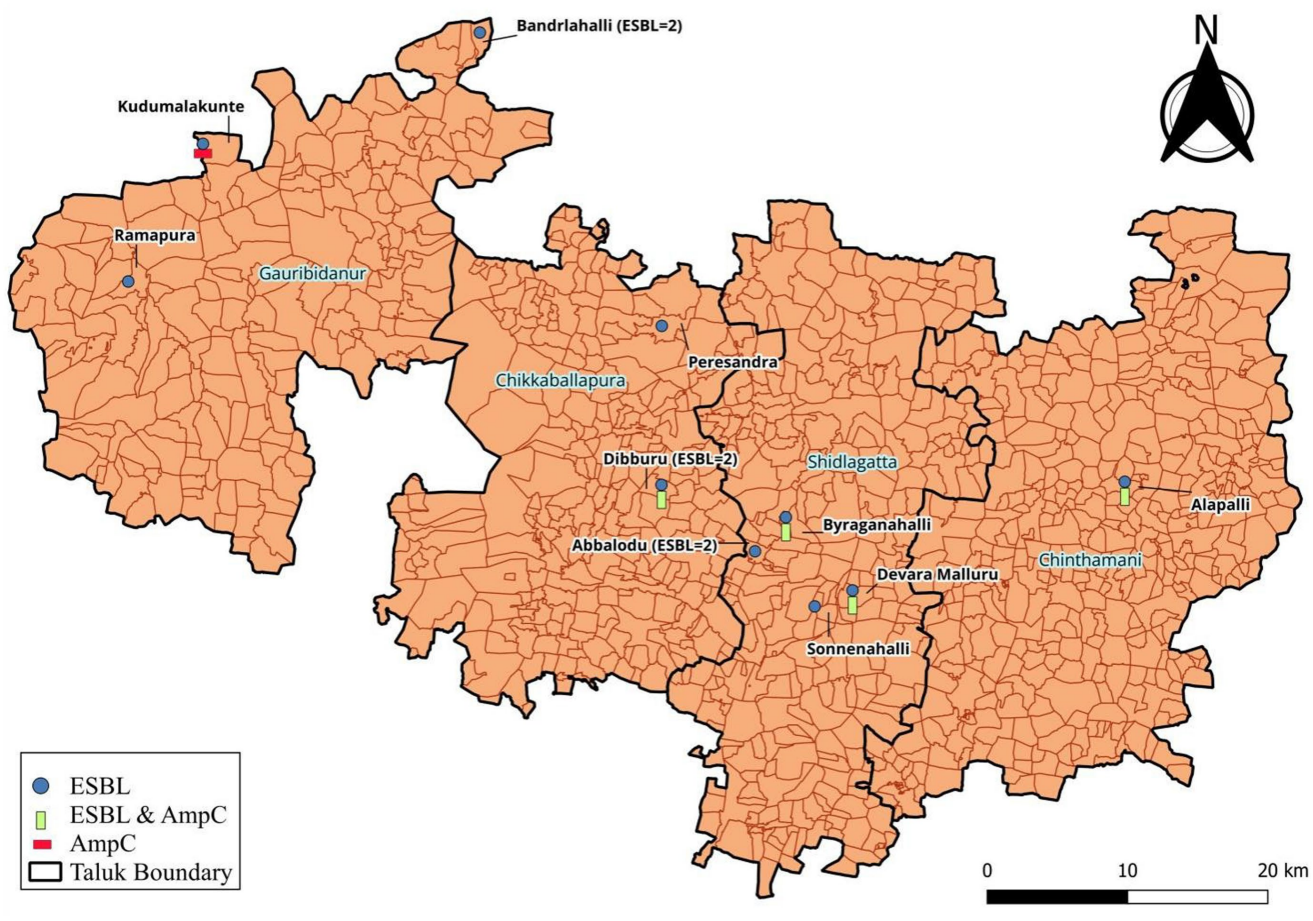
Spatial distribution of ESBL- and AmpC-producing *E. coli* isolates across different taluks of Chikkaballapura district, Karnataka, India. Blue circles indicate ESBL-positive isolates, green markers represent isolates co-harboring ESBL and *AmpC* genes, and red markers denote AmpC-positive isolates. Taluk boundaries are delineated, and sampling locations are labeled to illustrate the geographic clustering and spread of β-lactamase-producing *E. coli* within the study area.

PCR screening of all the 45 isolates revealed that 18 *E. coli* isolates had β-lactamase genes of which, *bla*_SHV_ was detected in 13/45 isolates (28.8%) followed by *bla*_TEM_ in 12 (26.6%) and *bla*_CTX-M_ in nine isolates (20%). The *AmpC* gene was present in five isolates (11.11%) confirming both ESBL and AmpC producers (Figure 1). Multiple ESBL determinants were identified in a subset of isolates with few showing both ESBL and *AmpC* genes (Supplementary Figures 1B–D).

### 3.3 *In vitro* AST profile

**Figure 2:**
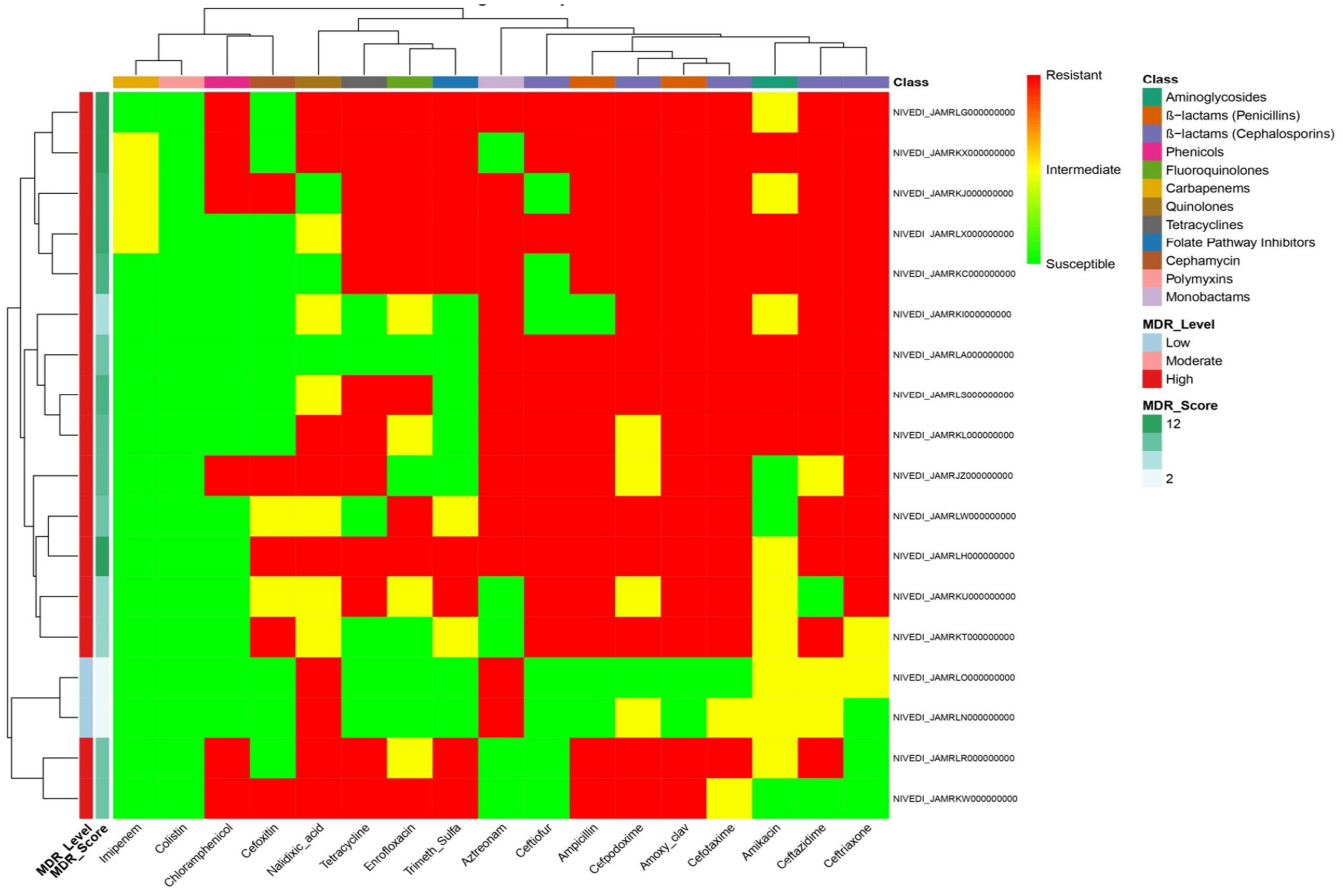
*In vitro* AST heatmap of *E. coli* isolates. Rows represent isolates and columns denote antibiotics clustered by hierarchical analysis; colors indicate susceptible (green), intermediate (yellow), and resistant (red) phenotypes. Most isolates exhibited high MDR scores with extensive resistance to β-lactams, fluoroquinolones, and tetracyclines.

AST profiling of the 18 *E. coli* isolates against 17 antibiotics representing 12 antimicrobial classes revealed high resistance rates against amoxy-clavulanic acid (88.9%), ampicillin (83.3%), cefotaxime (83.3%) and third-generation cephalosporins including cefpodoxime, ceftazidime, aztreonam and ceftriaxone (each 72.2%). Moderate resistance was noted for tetracycline (66.7%), ceftiofur (61.1%) and fluoroquinolones such as enrofloxacin and nalidixic acid (50% each) whereas chloramphenicol and amikacin exhibited lower resistance levels (33.3%). Imipenem showed the highest efficacy with 83.3% susceptibility and all isolates remained fully susceptible to colistin (100%). Phenotypic ESBL production was detected in 14/18 isolates (77.8%) and AmpC in 5/18 isolates (27.8%) including four co-producers (22.2%). All isolates showed reduced susceptibility to cefotaxime and ceftriaxone (100% R/I) while ceftazidime (83.3%) and cefpodoxime (94.4%) also displayed high non-susceptibility. Aztreonam similarly showed 94.4% resistance/intermediate response consistent with ESBL/AmpC activity. The isolates exhibited an MDR phenotype dominated by resistance to β-lactams, quinolones and tetracyclines (Figure 2).

### 3.4 Whole genome sequence analyses

#### 3.4.1 ARG resistome profile

**Figure 3:**
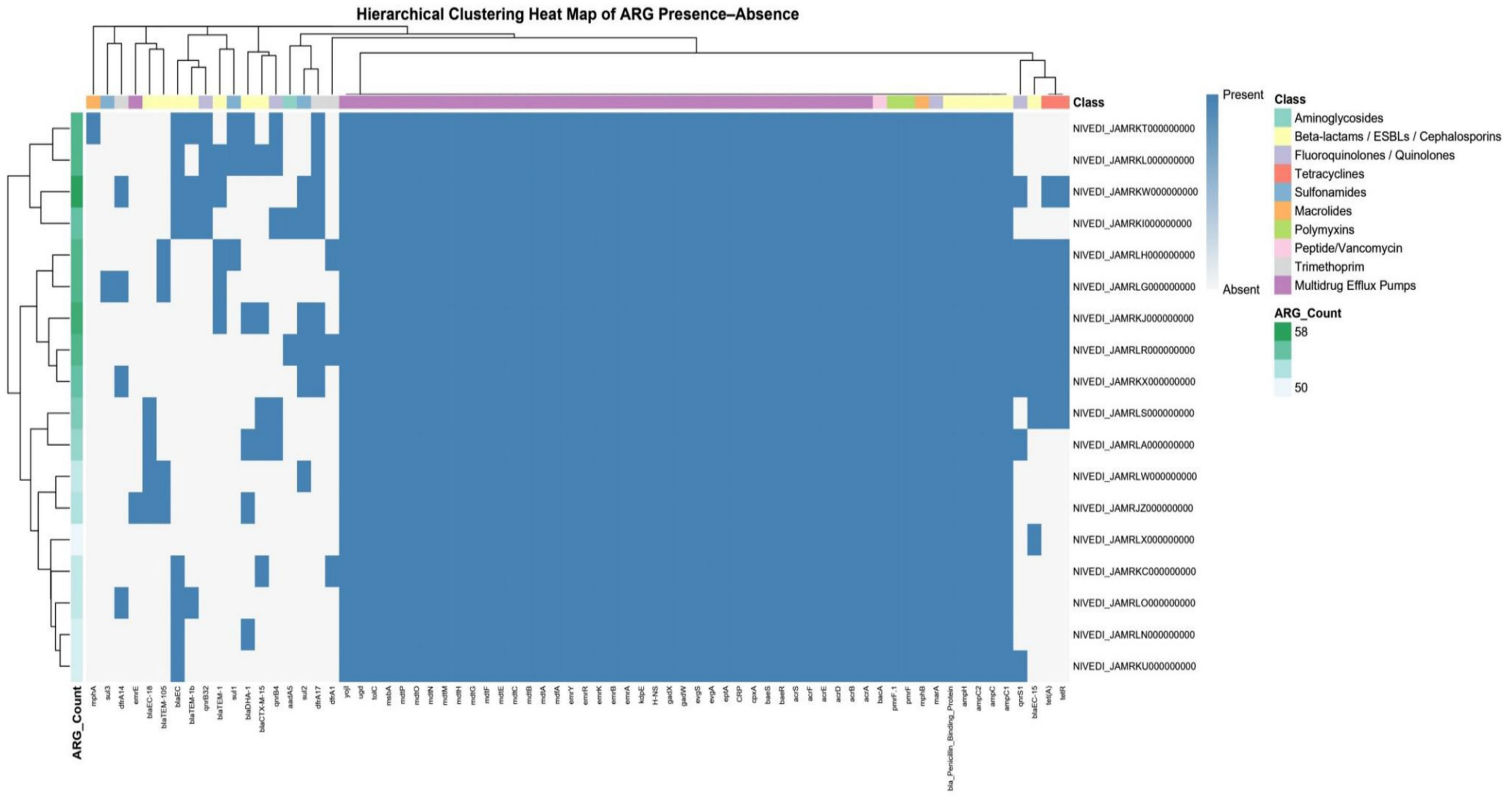
Genotypic resistome heatmap of *E. coli* isolates. Rows represent isolates and columns denote ARGs, clustered hierarchically; blue indicates gene presence and white indicates absence with classes annotated above. All isolates harbor dense and overlapping ARG repertoires dominated by β-lactam, efflux, fluoroquinolone, and aminoglycoside determinants.

Across 18 poultry *E. coli* isolates revealed 100% presence of multidrug efflux and regulatory systems including *acrA, B, D, E, F, S, emrA, B, mdfA, baeR, S, mdt* clusters and *marA* along with chromosomal β-lactam associated genes (*ampC, ampC1, ampC2, ampH*) and stress-response loci (*bacA, baeR, baeS*). The aminoglycoside gene *aadA5* was present in only two isolates (11.1%). Trimethoprim-resistance genes were more common with *dfrA1* in 4/18 (22.2%), *dfrA14* in 5/18 (27.8%) and *dfrA17* in 6/18 (33.3%) with combined prevalence of 11/18 (61.1%). Sulfonamide genes (*sul1/sul2/sul3*) were identified in 6/18 isolates (33.3%) predominantly being *sul2.* Plasmid-mediated quinolone resistance genes (*qnrS1, qnrB4, qnrB32*) were noted in 5/18 isolates (27.8%). Among β-lactamases ESBL bla_CTX-M-15_ was present in 5/18 (27.8%), *AmpC bla*_DHA-1_ in 6/18 (33.3%), *bla*_EC_ family variants in 11/18 (61.1%) and *bla*_TEM_ alleles (*bla*_TEM-1_, *bla*_TEM-1b_, *bla*_TEM-105_) collectively in 6/18 isolates (33.3%). Tetracycline-resistance genes *tet(A)/tetR* appeared in 4/18 isolates (22.2%) while macrolide genes *mphA/B* in 3/18 (16.7%) (Figure 3). Notably, key ARG patterns showed lineage-level associations with β-lactamase genes (*bla*_CTX-M-15_, *bla*_TEM_ variants) and PMQR determinants frequently co-occurring in isolates belonging to phylogroups A and B1 and associated with IncF/IncHI plasmid backgrounds indicating structured distribution of resistance-associated features within dominant commensal lineages rather than direct evidence of dissemination.

#### 3.4.2 Virulence gene distribution

**Figure 4:**
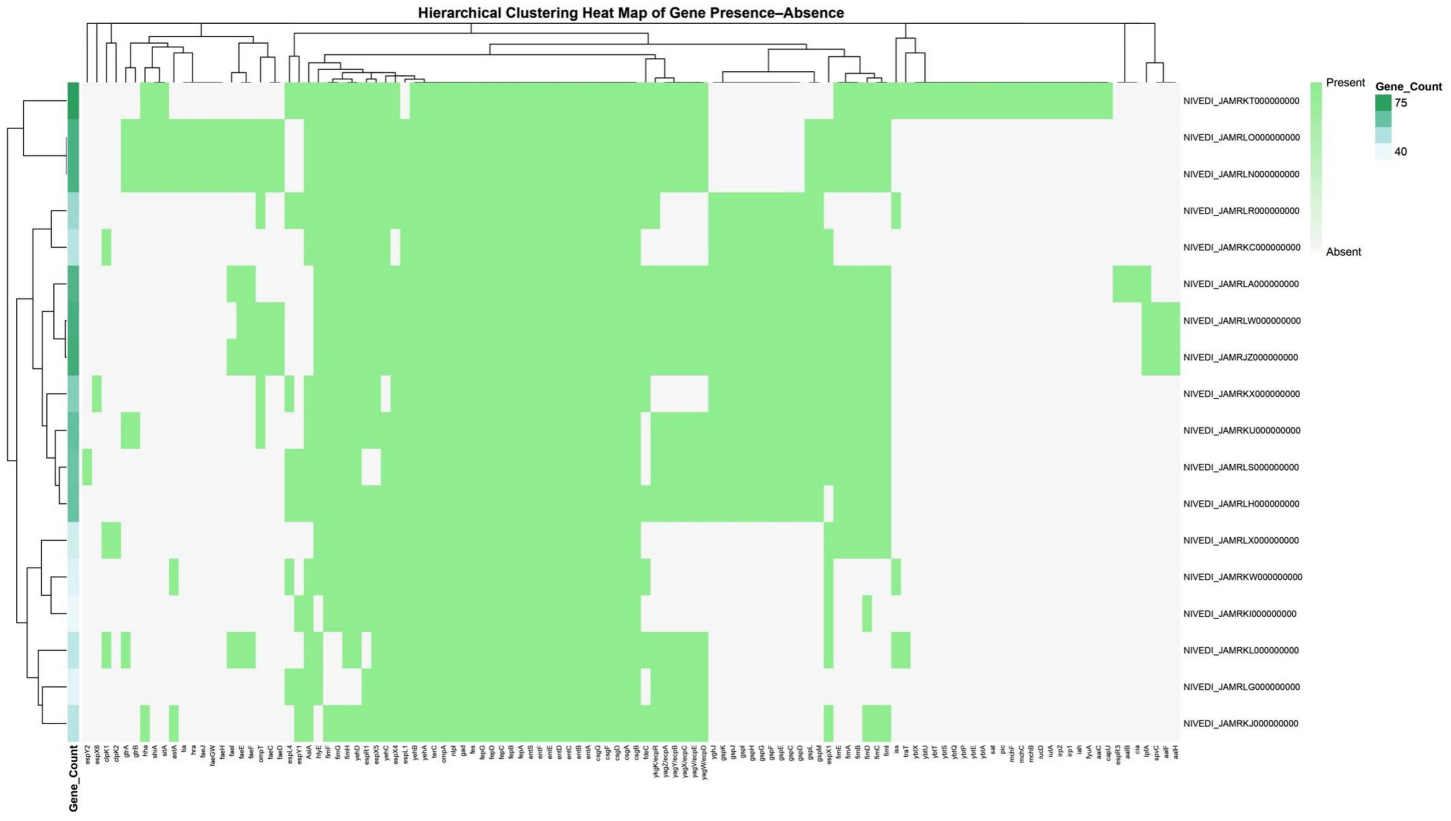
Virulence gene heatmap of *E. coli* isolates. Rows represent isolates and columns denote virulence-associated genes, hierarchically clustered; green indicates gene presence and white indicates absence.

Screening of 114 virulence-associated genes revealed a diverse virulence repertoire among isolates. Adhesion and colonization operons are *csgA csgA, B, D, F, G* (curli) and *fimA, B, C, D, E, F, G, H, I* (type 1 fimbriae) were widely conserved. Iron acquisition systems including *entA, B, C, D, E, F, S, fep/fes* and yersiniabactin genes (*ybtA, ybtE, ybtP, ybtQ, ybtS, ybtT, ybtU, ybtX*) were identified in multiple isolates. Secretion system genes (*gspC, D, E, F, G, H, I, J, K, L, M*) and stress-response determinants (*ompT, gad, hlyE, shiA, iss*) showed variable presence. Adherence and fimbrial determinants such as *lpfA, faeA, C, E, F, I, J* and the *ecp* (*yag*) pilus cluster occurred in selected isolates reflecting varied virulence. These findings suggest both ExPEC-like and APEC-like virulence traits circulating within poultry-associated *E. coli* (Figure 4). Importantly, isolates harboring enriched virulence gene profiles (*fim, csg, ybt* clusters) often overlapped with those carrying higher ARG burdens indicating co-localization of resistance and virulence traits within the same genomic backgrounds.

#### 3.4.3 Biofilm-associated genes

**Figure 5:**
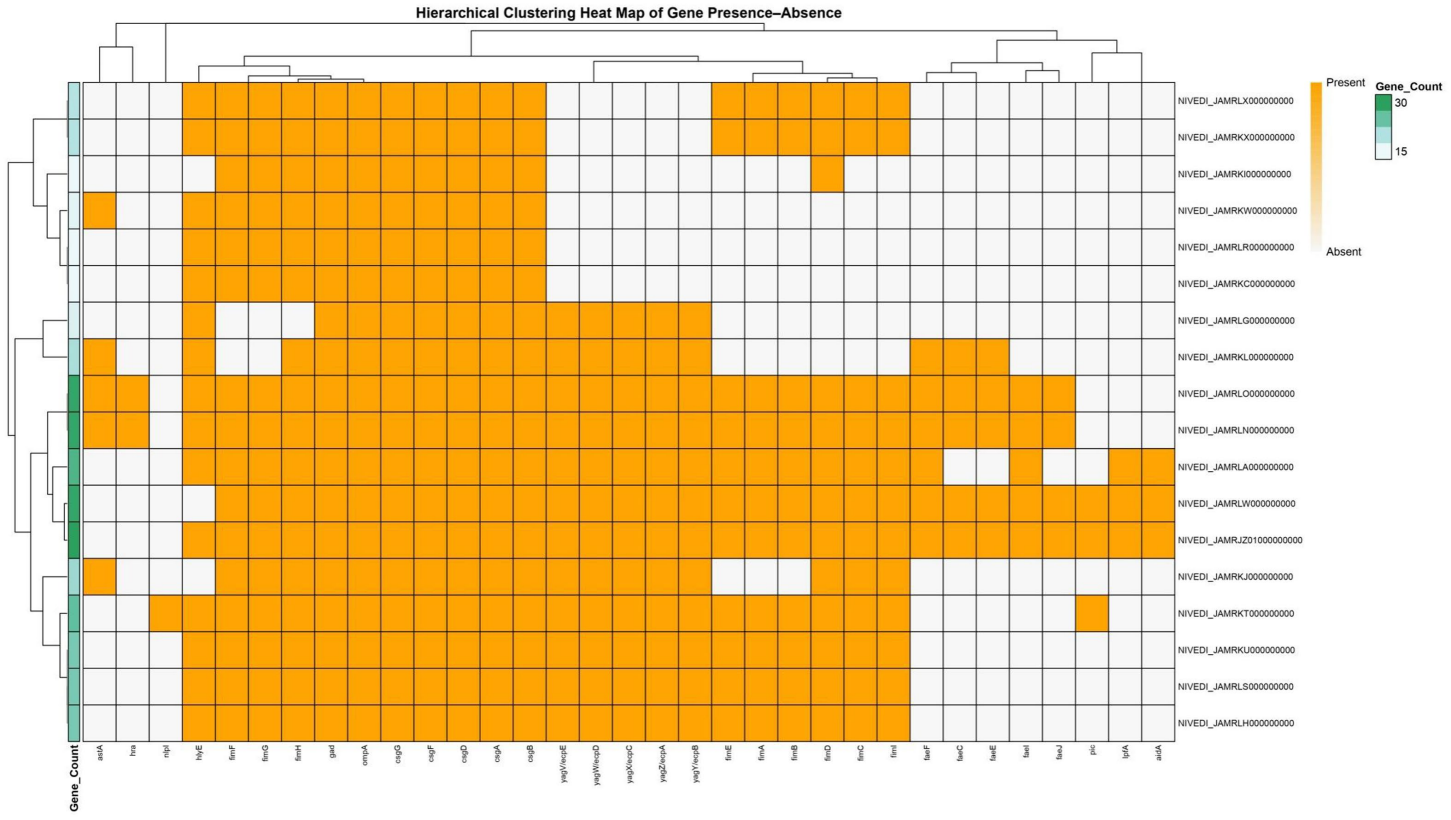
Biofilm-associated genes in *E. coli* isolates. Rows represent isolates and columns denote selected virulence and fitness genes; orange indicates gene presence and white indicates absence of genes with hierarchical clustering.

A total of 34 biofilm-associated genes were identified across the 18 *E. coli* isolates in various combinations. All the isolates had curli operon (*csgA, B, D, F, G*) indicating initial surface adhesion capability. The type 1 fimbrial cluster (*fimA, B, C, D, E, F, G, H, I*) was widely distributed among isolates harboring all major fimbrial components essential for biofilm establishment. The *E. coli* common pilus (*ecp*) locus genes (*yagZ, Y, X, W, V/ecpA, B, C, D, E*) were variably detected. Core outer membrane and structural genes such as *ompA* and *nlpI* were present in most isolates. Additional adherence and virulence-linked genes *faeA, C, E, F, I, J, lpfA*, *gad, hlyE, astA, hra* and *aidA* were observed in select genomes (Figure 5). The widespread presence of biofilm-associated genes particularly curli and type 1 fimbriae overlapped with isolates carrying multiple resistance genes and plasmids indicating that biofilm formation may contribute to the environmental persistence and stability of MDR phenotypes.

#### 3.4.4 Plasmid replicon profiling

Plasmid analysis revealed a heterogeneous plasmidome dominated by IncF-type replicons particularly IncFII and IncFIB which were among the most frequently detected across genomes. IncHI1A/IncHI1B plasmids were also common and often co-carried multiple ARGs such as *qnrS1, tet(A), bla*_TEM-1B_ and *sul2.* Several strains harbored high plasmid loads (up to 11 plasmids), suggesting increased genomic complexity associated with plasmid content. Col-type plasmids including Col (pHAD28), ColRNAI, Col156 and Col (MG828) were widely distributed and frequently paired with IncF replicons. The p0111-family plasmids appeared in many isolates and consistently co-occurred with MDR signatures. Less frequent replicons such as IncX1/X4, IncI1-I (*α*), IncB/O/K/Z and pENTAS02 contributed additional diversity. The plasmid landscape reflects a mosaic of large MDR-associated IncF/IncHI plasmids and widespread small Col-elements (Table 1). Plasmid replicon analysis further revealed that IncF and IncHI plasmids were consistently associated with major ARG classes particularly ESBL genes, indicating association with resistome patterns across diverse sequence types and phylogroups. However, these associations represent co-occurrence patterns within individual isolates and do not alone provide direct evidence of plasmid-mediated transmission between strains without detailed plasmid sequence comparison.

**Table 1:** Plasmid replicon profiles, CRISPR-Cas systems, and *in silico* serotype characterization (O- and H-antigens) of the 18 *E. coli* isolates showing the number of CRISPR arrays, Cas subtype, and corresponding O- and H-serogroups.

Sl. no	Isolate ID	Plasmids	CRISPR-Cas		Serotypes			
			No. of CRISPR arrays	Cas type/subtype	O-type gene	O-serotype	H-type gene	H-serotype
1	NIVEDI_JAMRLX000000000	pENTAS02, pSL483, IncFIB(K), IncFII, pXuzhou21	1	Type I-E	*–*	–	*fliC*	H4
2	NIVEDI_JAMRLW000000000	IncFIB(AP001918), IncFII(pHN7A8), IncFII(pSE11), Col(pHAD28), IncFII(pRSB107)	1	Type I-E	*wzm*	O8	*fliC*	H7
3	NIVEDI_JAMRLS000000000	p0111, ColRNAI, Col(pHAD28), ColpVC	1	Type I-E	*wzx*	O118/O151	*fliC*	H29
4	NIVEDI_JAMRLR000000000	IncHI1B(R27)_R27, IncHI1A, p0111	1	Type I-E	*wzt / wzy*	O8	*fliC*	H11
5	NIVEDI_JAMRLO000000000	-	2	Type I-E	*wzy / wzx*	O69	*fliC*	H38
6	NIVEDI_JAMRLN000000000	-	1	Type I-E	*wzx / wzy*	O69	*fliC*	H38
7	NIVEDI_JAMRLH000000000	p0111	3	Type I-E	*wzy / wzx*	O130	*fliC*	H26
8	NIVEDI_JAMRLG000000000	p0111, IncP1_3	1	Type I-E	*wzx / wzm*	O176 / O62	*fliC*	H11
9	NIVEDI_JAMRLA000000000	IncB/O/K/Z_2, IncHI1A, IncHI1B(pNDM-CIT), IncFII(pCoo), ColRNAI	2	Type I-E	*wzy / wzx*	O132	*fliC*	H28
10	NIVEDI_JAMRKX000000000	p0111, IncX4_2	Not detected	-	*wzx / wzy*	O158	*fliC*	H32
11	NIVEDI_JAMRKW000000000	p0111, IncHI1B(pNDM-CIT), Col(pHAD28)	1	Type I-E	*–*	–	*fliC*	H11
12	NIVEDI_JAMRKU000000000	IncHI1B(pNDM-CIT), p0111	1	Type I-E	*–*	–	*fliC*	H10
13	NIVEDI_JAMRKT000000000	IncFII, IncFIB(AP001918), ColRNAI, ColRNAI, Col(MG828)	Not detected	-	*wzx / wzy*	O154	*fliC*	H19
14	NIVEDI_JAMRKL000000000	IncI1-I(Alpha), IncFII, IncX1, Col(pHAD28)	3	Type I-E	*wzy / wzx*	O127	*fliC*	H26
15	NIVEDI_JAMRKJ000000000	IncX1	1	Type I-E	*wzy / wzx*	O96	*fliC*	H5
16	NIVEDI_JAMRKI000000000	p0111, IncHI1B(pNDM-CIT)	1	Type I-E	*wzy / wzx*	O96	*fliC*	H5
17	NIVEDI_JAMRKC000000000	pENTAS02, IncX1	Not detected	-	*wzx / wzy*	O40	*fliC*	H10
18	NIVEDI_JAMRJZ000000000	IncFII(pRSB107), IncHI1A, IncHI1B(pNDM-CIT), IncFII(pSE11), IncFIB(AP001918), Col(pHAD28), Col440II, Col(pHAD28), Col156, ColRNAI, Col(MG828)	1	Type I-E	*wzy / wzx / wzm*	O18 / O18ac / O8	*fliC*	H7

#### 3.4.5 CRISPR-Cas system profiling

CRISPR-Cas analysis of the 18 poultry *E. coli* genomes showed 15 isolates (83.3%) with identifiable CRISPR elements predominantly belonging to the Type I-E CRISPR-Cas system. Most CRISPR-positive genomes harbored one or more high-confidence arrays (evidence-level 4) accompanied by a single well-organized Cas operon. Notably, CRISPR-Cas loci frequently coexisted with multiple plasmid replicon types including IncF, IncHI1, IncX1, IncI1, p0111 and Col plasmids indicating co-occurrence of CRISPR systems with MGE within the same isolates rather than functional interaction. These 15 CRISPR-positive isolates also encoded a wide array of AMR genes with dominant determinants of β-lactamases (*bla*_CTX-M_ family and plasmid AmpC variants), aminoglycoside (*aadA*) and fluoroquinolone resistance genes (*qnrS/qnrB*). Virulence profiling revealed enrichment for adhesion/biofilm-associated genes (*csg, fim, ecpA*), motility genes (*fli/mot*), quorum-sensing regulator *(luxS)* and global stress regulators (*rpoS, hfq*), supporting enhanced host adaptation and persistence (Table 1). Interestingly, CRISPR-Cas–positive isolates also carried multiple plasmid types and ARGs; however, this observation reflects isolate-level co-occurrence and does not provide evidence regarding the functional activity of CRISPR systems or their influence on gene acquisition.

#### 3.4.6 MLST analysis

MLST analysis using the Achtman scheme revealed substantial sequence type (ST) diversity among the 18 *E. coli* isolates. Allelic profiles of housekeeping genes (*adk, fumC, gyrB, icd, mdh, purA* and *recA*) showed wide variation particularly in *icd* and *purA* indicating genetic heterogeneity among isolates. A total of 12 distinct STs were identified with ST48 being the most common (NIVEDI_JAMRKW000000000, NIVEDI_JAMRLR000000000, NIVEDI_JAMRLS00000000) and ST3107 (NIVEDI_JAMRLN000000000, NIVEDI_JAMRLO000000000) each represented by two isolates. Several isolates showed unique STs such as ST10, ST165, ST206, ST216, ST226, ST2705, ST2936 and ST5834. Four isolates did not match an exact sequence type and were assigned to the nearest STs (297, 3,576, 6,856, and 3,576) (Table 2). Despite high sequence type diversity, ARG and virulence profiles showed consistent enrichment within phylogroups A and B1, indicating that these commensal lineages act as stable genomic platforms for accumulation of resistance and adaptive traits.

**Table 2:** Achtman MLST profiles of the 18 *E. coli* isolates showing allelic combinations of the seven housekeeping genes (*adk, fumC, gyrB, icd, mdh, purA, and recA*) and the corresponding STs or nearest ST assignment.

Sl. no	Isolate ID	Achtman MLST scheme	ST	*adk*	*fumC*	*gyrB*	*icd*	*mdh*	*purA*	*recA*
1	NIVEDI_JAMRLX000000000	ecoli_achtman_4	216	10	11	57	8	7	18	6
2	NIVEDI_JAMRLW000000000	ecoli_achtman_4	Nearest ST: 3576	563	29	32	16	9	8	2
3	NIVEDI_JAMRLS000000000	ecoli_achtman_4	48	6	11	4	8	8	8	2
4	NIVEDI_JAMRLR000000000	ecoli_achtman_4	48	6	11	4	8	8	8	2
5	NIVEDI_JAMRLO000000000	ecoli_achtman_4	3,107	10	11	5	8	7	219	2
6	NIVEDI_JAMRLN000000000	ecoli_achtman_4	3,107	10	11	5	8	7	219	2
7	NIVEDI_JAMRLH000000000	ecoli_achtman_4	5,834	10	27	4	10	12	8	6
8	NIVEDI_JAMRLG000000000	ecoli_achtman_4	Nearest ST: 6856	6	11	4	732	8	514	2
9	NIVEDI_JAMRLA000000000	ecoli_achtman_4	Nearest ST: 297	6	65	32	26	9	8	2
10	NIVEDI_JAMRKX000000000	ecoli_achtman_4	2,936	10	11	4	8	8	2	2
11	NIVEDI_JAMRKW000000000	ecoli_achtman_4	48	6	11	4	8	8	8	2
12	NIVEDI_JAMRKU000000000	ecoli_achtman_4	2,705	10	11	4	10	7	8	2
13	NIVEDI_JAMRKT000000000	ecoli_achtman_4	10	10	11	4	8	8	8	2
14	NIVEDI_JAMRKL000000000	ecoli_achtman_4	165	10	27	5	10	12	8	2
15	NIVEDI_JAMRKJ000000000	ecoli_achtman_4	206	6	7	5	1	8	18	2
16	NIVEDI_JAMRKI000000000	ecoli_achtman_4	206	6	7	5	1	8	18	2
17	NIVEDI_JAMRKC000000000	ecoli_achtman_4	226	10	27	5	8	8	7	2
18	NIVEDI_JAMRJZ000000000	ecoli_achtman_4	Nearest ST: 3576	563	29	32	16	9	8	2

#### 3.4.7 MGE analysis

The MGE analysis observed 17 distinct MGEs and dominant being MITEEc1 (100%). Other common MGEs included IS609 (66.7%), ISEc1 (66.7%), ISEc5 (55.6%), IS102 (55.6%) and IS30 (50%) reflecting genomic diversity. The clinically important IS26 was present in 44.4% of strains and was observed in isolates carrying key ARGs such a*s bla*_CTX-M-15_*, bla*_TEM-1B_, *tet(A), sul2, sul3, dfrA1/14/17, qnrS1/S13, qnrb4/7* and *aadA5.* Isolates harboring multiple MGEs showed the highest accumulation of MDR clusters. Virulence determinants including *AslA, csgA, fimH, terC, ompT, yghJ* and *yehABC(D)* were consistently detected in MGE-rich genomes (Table 3). The co-occurrence of MGEs such as IS26 with resistance and virulence genes suggests potential genetic association between these elements; however, this reflects isolate-level patterns and does not provide direct evidence of HGT or physical linkage. Furthermore, contig-level co-localization or physical linkage of MGEs with specific resistance or virulence genes was not resolved in the present analysis.

**Table 3:** Distribution of MGEs, AMR genes, virulence determinants, and corresponding phenotypic resistance profiles among the 18 *E. coli* isolates.

Sl. no	Isolate ID	MGE	Resistance genes	Virulence genes	Phenotype
1	NIVEDI_JAMRLX000000000	IS903, MITEEc1, Tn6024, ISEc1, ISEc26, IS102, IS26, IS421, cn_7361_IS26	*-*	*hlyE, csgA, clpK1, terC, nlpI, gad, clpK2, yehD, yehB, yehA, yehC, fimH*	-
2	NIVEDI_JAMRLW000000000	IS609, IS609, ISEc5, MITEEc1, IS682, IS3, IS679	*-*	*lpfA, aalH, terC, yghJ, nlpI, terC, aalF, ompT, fdeC, gad, gad, fimH, faeE, faeF, faeC, yehD, yehB, yehA, yehC*	-
3	NIVEDI_JAMRLS000000000	ISEc1, ISEc5, MITEEc1	*tet(A)*	*gad, yghJ, espY2, terC, nlpI, terC, csgA, fimH, hlyE, AslA, gad, yehD, yehB, yehA, yehC*	Tetracycline, doxycycline
4	NIVEDI_JAMRLR000000000	MITEEc1, IS100, IS679, IS421, ISEc1, ISEc5, IS30, ISEc38, IS3, IS102	*sul2, dfrA17, tet(A), qnrS1, aadA5*	*AslA, gad, terC, ompT, iss, nlpI, terC, hlyE, fdeC, fimH, yghJ, csgA, yehD, yehB, yehA, yehC*	Sulfamethoxazole, trimethoprim, tetracycline, doxycycline, ciprofloxacin, spectinomycin, streptomycin
5	NIVEDI_JAMRLO000000000	ISEc1, MITEEc1, IS609, ISEc5, IS30, IS682, IS640	*sitABCD*	*sitA, tia, shiA, terC, fimH, gad, hha, AslA, faeC, faeJ, hra, terC, nlpI, fdeC, tia, shiA, faeF, ompT, gad, yehD, yehB, yehA, yehC, faeC, faeE, faeI, faeJ, faeGW, faeF, faeH, faeD, astA, csgA, hlyE, hha*	Hydrogen peroxide
6	NIVEDI_JAMRLN000000000	ISEc1, IS609, MITEEc1, ISEc5, IS30, IS640, IS682, IS421,	*sitABCD*	*sitA, astA, faeF, terC, hra, faeJ, tia, shiA, fdeC, terC, nlpI, gad, fimH, ompT, AslA, gad, shiA, tia, faeC, hha, csgA, hlyE, faeE, faeI, faeJ, faeGW, faeF, faeH, yehD, yehB, yehA, yehC, faeD*	Hydrogen peroxide
7	NIVEDI_JAMRLH000000000	MITEEc1, IS609, ISEc5, IS102, ISEc1, IS629, cn_18841_IS102, cn_22381_IS102	*dfrA1, blaTEM-1B, qnrS1, tet(A), dfrA1, dfrA1*	*gad, gad, AslA, csgA, hlyE, fimH, nlpI, yghJ, fdeC, terC*	Trimethoprim, cephalothin, piperacillin, amoxicillin, ticarcillin, ampicillin, ciprofloxacin, tetracycline, doxycycline
8	NIVEDI_JAMRLG000000000	MITEEc1, IS102, ISEc1, cn_51534_IS102, cn_2244_ISEc1	*tet(A), dfrA14, sul3, qnrS1, blaTEM-1B*	*AslA, nlpI, terC, gad, csgA, hlyE, gad, terC, yehD, yehB, yehA, yehC*	Doxycycline, tetracycline, trimethoprim, sulfamethoxazole, ciprofloxacin, piperacillin, ticarcillin, cephalothin, ampicillin, amoxicillin
9	NIVEDI_JAMRLA000000000	MITEEc1, IS609, ISEc5, IS5, IS4, IS100, IS3, cn_1811_ISEc5	*qnrS1, blaCTX-M-15*	*cia, nlpI, lpfA, aalB, faeI, faeF, yghJ, terC, fdeC, csgA, hlyE, gad, terC, fimH, gad, yehD, yehB, yehA, yehC*	Ciprofloxacin, cefepime, cefotaxime, amoxicillin, ceftriaxone, aztreonam, ampicillin, piperacillin, ceftazidime, ticarcillin
10	NIVEDI_JAMRKX000000000	IS3, MITEEc1, ISEc1, IS609, IS26, ISVsa3, IS5075, IS421, IS903, IS102, cn_1496_ISEc1	*dfrA14, tet(A), qnrS1*	*yghJ, yghJ, terC, terC, nlpI, terC, fdeC, yehD, gad, ompT, AslA, csgA, hlyE, yehA, yehB, fimH*	Trimethoprim, doxycycline, tetracycline, ciprofloxacin
11	NIVEDI_JAMRKW000000000	IS5, MITEEc1, IS3, IS102, ISEc5, ISEc1, IS30, IS26, cn_1670_ISEc1, cn_3458_IS26	*blaTEM-1B, sul2, qnrS13, dfrA14, tet(A)*	*iss, iss, fimH, fimH, terC, nlpI, hlyE, gad, astA, csgA, fdeC, terC, gad, AslA, yehD, yehB, yehA, yehC*	Ampicillin, piperacillin, ticarcillin, amoxicillin, cephalothin, sulfamethoxazole, ciprofloxacin, trimethoprim, doxycycline, tetracycline
12	NIVEDI_JAMRKU000000000	IS609, MITEEc1, ISEc1, IS30, IS26, IS102	*-*	*yghJ, terC, yghJ, terC, yehD, yehB, yehA, yehC, AslA, hlyE, csgA, nlpI, gad, fimH, ompT, gad*	Ciprofloxacin
13	NIVEDI_JAMRKT000000000	MITEEc1, ISEc1, IS6100, IS26, IS629, IS30, ISEc1, IS421, cn_3319_IS26, cn_10539_IS629	*qnrB4, qacE, mph(A), sul1, blaDHA-1, sitABCD, dfrA17*	*traT, sitA, hha, hha, terC, nlpI, terC, gad, AslA, fdeC, fimH, irp2, fyuA, iss, iss, csgA, hlyE, gad, hha, aaiC, terC, capU, mchF, mchB, mchC, iha, yehD, yehB, yehA, yehC, sat, shiA, pic*	Ciprofloxacin, ethidium bromide, cetylpyridinium chloride, chlorhexidine, benzylkonium chloride, spiramycin, erythromycin, telithromycin, azithromycin, sulfamethoxazole, piperacillin+tazobactam, ampicillin, cefoxitin, cefotaxime, piperacillin, ceftazidime, ticarcillin+clavulanic acid, ticarcillin, amoxicillin+clavulanic acid, ampicillin+clavulanic acid, amoxicillin, hydrogen peroxide, trimethoprim
14	NIVEDI_JAMRKL000000000	MITEEc1, IS609, ISEc5, ISEc1, IS30, IS421, IS3	*qnrS1, blaCTX-M-15*	*faeE, faeI, faeF, traT, yehD, yehB, yehA, yehC, terC, nlpI, terC, clpK1, fdeC, iss, iss, gad, gad, fimH, csgA, AslA, hlyE*	Ciprofloxacin, ticarcillin, cefepime, aztreonam, cefotaxime, piperacillin, ceftriaxone, ampicillin, amoxicillin, ceftazidime
15	NIVEDI_JAMRKJ000000000	IS100, MITEEc1, ISEc1, IS609, ISEc1, IS26, IS102, cn_2244_ISEc1	*tet(A), qnrS13*	*hha, fimH, gad, gad, csgA, terC, nlpI, terC, AslA, fdeC, astA, yehD, yehB, yehA, yehC*	Doxycycline, tetracycline, ciprofloxacin
16	NIVEDI_JAMRKI000000000	IS100, MITEEc1, IS3, IS609, ISEc1, IS5, IS30, IS26, IS26, IS102, ISVsa3, MITEEc1, cn_2244_ISEc1, cn_1629_IS102	*dfrA17, sul2, aadA5, qnrB7*	*gad, csgA, AslA, terC, fimH, terC, nlpI, yehD, yehB, yehA, yehC*	Trimethoprim, sulfamethoxazole, streptomycin, spectinomycin, ciprofloxacin
17	NIVEDI_JAMRKC000000000	IS100, IS5, IS421, ISEc1, MITEEc1, Tn6024, ISEc1, ISEc5, IS609, cn_3155_ISEc1	*-*	*yghJ, terC, nlpI, yghJ, yehD, yehB, yehA, yehC, AslA, gad, hlyE, csgA, clpK1, terC, fimH, gad*	-
18	NIVEDI_JAMRJZ000000000	IS609, ISEc5, MITEEc1, IS679, IS4, IS100, IS421, IS629	*-*	*terC, nlpI, csgA, hlyE, yghJ, terC, fimH, ompT, lpfA, gad, fdeC, faeF, faeE, faeC, aalF, aalH, faeI, yehD, yehB, yehA, yehC*	-

#### 3.4.8 Serotype distribution

*In silico* analysis of O and H antigen genes revealed diverse serotypes among the 18 *E. coli* isolates. O-antigen determinants (*wzx, wzy, wzm, wzt*) corresponded to 14 distinct O-serotypes (O8, O18/O18ac/O8, O40, O69, O96, O118/O151, O127, O130, O132, O154, O158 and O176/O62). The identifiable O-type genes were absent in three isolates were categorized as O-non-typeable (ONT). H-typing showed consistent detection of *fliC* in all isolates with H4, H5, H7, H10, H11, H19, H26, H28, H29, H32 and H38 as the major flagellar antigens. The repeated presence of O8, O69:H38 and O96:H5 associated serotypes suggests possible clonal relationships among isolates commonly associated with APEC or ExPEC infections (Table 1).

#### 3.4.9 CH typing (*fumC-fimH* alleles)

CH typing based on *fumC* and *fimH* allelic combinations identified 12 distinct CH types among the 18 *E. coli* isolates indicating high genetic diversity. The *fumC11* allele was predominant (detected in 61% of isolates), followed by *fumC7, fumC27, fumC29* and *fumC65*. The *fimH* alleles were diverse with *fimH*23, fimH54 and *fimH*68 being the most frequent variants. Several isolates shared the *fumC7-fimH23* and *fumC11-fimH54* combinations suggesting clonal relationships among commensal lineages. The presence of unique alleles like *fimH400, fimH1067* and *fimH3330* as novel or region-specific variants in poultry (Table 4).

**Table 4:** *In silico* CH-typing and clermont phylogroup assignment of the 18 *E. coli* isolates showing *fumC–fimH* allelic profiles, quadruplex PCR markers, supporting genes and concordance between clermont phylogroup and mash-based clustering.

SL. No	Isolate Id	CH typer allels		*In Silico* Clermont phylotyper						
				Quadruplex PCR assay				Supporting genes	phylogroup	Mash_group
		*fumC*	*fimH*	*arpA*	*chu*	*yjaA*	*TspE*4			
1	NIVEDI_JAMRLX000000000	*fumC*11	*fimH*69	+	−	−	−	*fdm, cfaB, ybgD*	A	A
2	NIVEDI_JAMRLW000000000	*fumC29*	*fimH*68	+	−	−	+	*fdm, trpAgpC, ybgD*	B1	B1
3	NIVEDI_JAMRLS000000000	*fumC*11	*fimH*400	+	−	−	−	*fdm, ybgD*	A	A
4	NIVEDI_JAMRLR000000000	*fumC*11	*fimH*54	+	−	+	−	*ybgD*	A	A
5	NIVEDI_JAMRLO000000000	*fumC*11	*fimH*41	+	−	−	−	*fdm, ybgD*	A	A
6	NIVEDI_JAMRLN000000000	*fumC*11	*fimH*41	+	−	−	−	*fdm, ybgD*	A	A
7	NIVEDI_JAMRLH000000000	*fumC*27	*fimH*54	+	−	+	−	*fdm, cfaB, ybgD*	A	A
8	NIVEDI_JAMRLG000000000	*fum*C11	*−*	+	−	+	−	*fdm, ybgD*	A	A
9	NIVEDI_JAMRLA000000000	*fumC*65	*fimH*38	+	−	−	+	*fdm, ybgD*	B1	B1
10	NIVEDI_JAMRKX000000000	*fumC*11	*fimH*1067	+	−	+	−	*fdm, ybgD*	A	A
11	NIVEDI_JAMRKW000000000	*fumC*11	*fimH*34	+	−	+	−	*fdm, ybgD*	A	A
12	NIVEDI_JAMRKU000000000	*fumC*11	*fimH*23	+	−	−	−	*fdm, ybgD*	A	A
13	NIVEDI_JAMRKT000000000	*fumC*11	*fimH*54	+	−	+	−	*fdm*	A	A
14	NIVEDI_JAMRKL000000000	*fumC*27	*fimH*3330	+	−	−	−	*fdm, ybgD*	A	A
15	NIVEDI_JAMRKJ000000000	*fumC*7	*fimH*23	+	−	+	−	*fdm*	A	A
16	NIVEDI_JAMRKI000000000	*fumC*7	*fimH*23	+	−	+	−	*fdm*	A	A
17	NIVEDI_JAMRKC000000000	*fumC*27	*fimH*54	+	−	−	−	*fdm, ybgD*	A	A
18	NIVEDI_JAMRJZ000000000	*fumC*29	*fimH*68	+	−	−	+	*fdm, trpAgpC, ybgD*	B1	B1

#### 3.4.10 Clermont phylotyping

*In silico* Clermont phylogrouping revealed that the majority of poultry *E. coli* isolates belonged to phylogroup A (15/18; 83.3%) reflecting predominantly commensal or low-virulence lineages common in avian reservoirs. A smaller cluster grouped into phylogroup B1 (3/18; 16.7%) which is typically associated with intestinal colonizers but may also indicate environmentally adapted strains. All isolates were *arpA*-positive (18/18; 100%), whereas *chuA* was absent in all strains confirming that none belonged to virulent B2/D lineages. The marker *yjaA* was detected in 8/18 isolates (44.4%) while *TspE4* was present in 3/18 (16.7%) supporting the separation of A and B1 groups. Supporting genes such as *fdm, ybgD, cfaB* and *trpA* and *gpC* were consistent with the predicted phylogroups and Mash clustering fully align with the Clermont assignments (Table 4). Despite high sequence type diversity, ARG and virulence profiles showed consistent enrichment within phylogroups A and B1 indicating that these commensal lineages act as stable genomic platforms for accumulation of resistance and adaptive traits.

### 3.5 Comparative phylogenomics and core-genome SNP-based phylogeny

**Figure 6:**
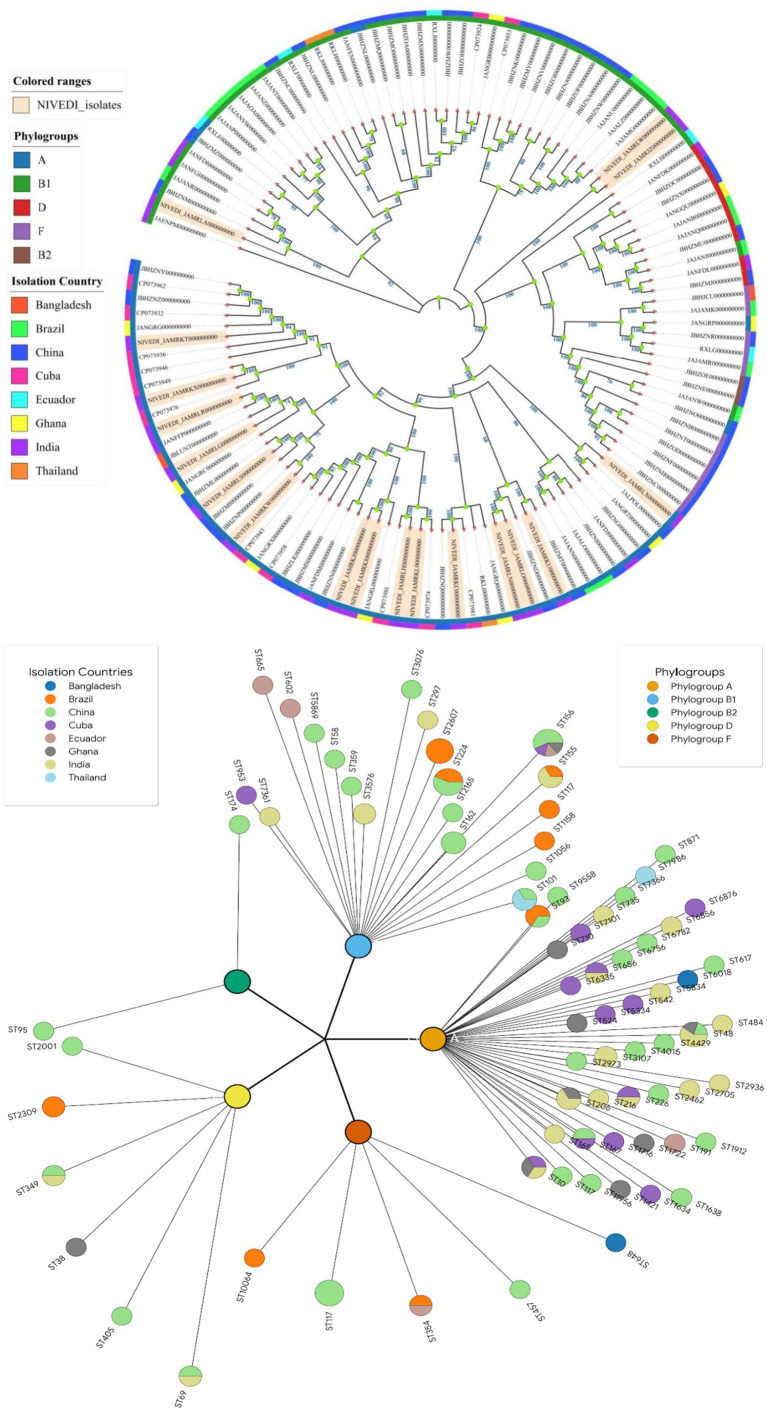
**(A)** Core genome SNP-based phylogenetic tree and **(B)** MLST radial network of *E. coli* isolates including NIVEDI strains and global reference genomes. The circular phylogenetic tree (left) was constructed from WGS-SNP alignments and visualized in iTOL with NIVEDI isolates highlighted in beige color. Outer color strips indicate Clermont phylogroups (A, B1, B2, D, and F) and country of origin. Bootstrap support values are shown at internal nodes. The radial network (right) displays STs from 123 poultry-associated *E. coli* genomes where nodes represent STs colored by phylogroup and sector-shaded by country of origin. Phylogroups A and B1 dominate the collection and include most Indian NIVEDI isolates reflecting clustering with globally disseminated commensal lineages, while phylogroups B2, D, and F are sparsely represented.

Core-genome SNP-based ML phylogeny of 123 *E. coli* genomes [18 NIVEDI isolates and 105 global poultry-associated references (Supplementary Table S1)] resolved robust clustering corresponding to phylogroups A, B1, B2, D, and F. The NIVEDI isolates segregated predominantly into two closely related clades within phylogroups A and B1 exhibiting short internal branch lengths consistent with limited core-genome divergence and indicating genetic relatedness among isolates rather than definitive evidence of localized circulation within local poultry populations. These NIVEDI clades were dominated by commensal-associated MLST sequence types (ST48, ST206, ST216, ST226, ST297, ST2705, ST2936, ST3107, ST3576, ST5834, and ST6856), indicating a restricted ST repertoire among the study isolates. In contrast, reference genomes from China (n-48) and Brazil (n-17) spanned broader phylogroup and ST diversity whereas genomes from Ghana, Cuba, and Ecuador formed distinct regionally structured clusters. Across the tree, commensal phylogroups (A and B1) remained clearly separated from extra intestinal lineages (B2, D, and F) with the latter being sparsely represented among poultry-derived genomes in this dataset (Figure 6).

Notably, NIVEDI isolates were interspersed with global reference genomes suggesting shared ancestry with widely distributed commensal lineages rather than a unique localized clonal expansion. Phylogenetic clustering further supports this observation where isolates sharing similar resistance and virulence profiles grouped within closely related clades indicating lineage-associated patterns of genomic features rather than random distribution; however, such clustering should be interpreted cautiously and does not alone confirm direct transmission in the absence of pairwise SNP distance analysis and temporal data. The observed grouping of isolates with similar resistance and virulence features reflects co-occurrence of these determinants at the isolate level; however, contig-level co-localization or physical linkage was not resolved in the present study. Additionally, no clear clustering of isolates based on village or block-level origin was observed, indicating the absence of evident spatial structuring within the sampled population.

### 3.6 PCA analysis

PCA successfully resolved the genetic and phenotypic architecture of the isolate with the resistome-phenotype analysis explaining 41.2% of total variance (PC1: 22.1%; PC2: 19.1%). Analysis of the virulome and biofilm profiles captured 45.29% of variance in the first two components (PC1: 24.52%; eigenvalue 30.22; PC2: 20.77%; eigenvalue 25.60) with cumulative variance reaching 76.69% across five components. PC1 was primarily driven by *bla*_CTX-M-15_, *bla*_TEM-1b_, and the *ybt* (Yersiniabactin) operon whereas PC2 was defined by *fae* fimbrial clusters and *qnr/sul* alleles. Visualizing the data through a plasma gradient palette revealed distinct clustering based on cumulative resistance complexity rather than random variation, identifying AmpC as a conserved core element while mobile elements drove MDR sub-populations (Supplementary Figure 2; Table 3). The PCA analysis provided an integrated view of genotype–phenotype relationships demonstrating that variation among isolates is primarily driven by combined effects of ARGs, virulence determinants, and MGEs, rather than individual features highlighting the integrated genomic architecture of MDR *E. coli*.

### 3.7 Statistical analysis

The prevalence of ESBL/AmpC-producing isolates was significantly higher than a baseline prevalence of 20% derived from previously reported lower-bound estimates in comparable poultry systems (Z = 3.46, *p* < 0.001). Resistance frequencies differed markedly across antimicrobial classes (χ^2^, *p* < 0.001) and all sequenced ESBL/AmpC isolates fulfilled the MDR definition, reflecting phenotype-enriched selection for WGS and not an estimate of MDR prevalence among all backyard poultry *E. coli*. ARG burden varied significantly between phylogroups (one-way ANOVA, *p* = 0.036), indicating lineage-specific resistance accumulation; however, given the unequal sample sizes across phylogroups, these results were considered exploratory. Pairwise comparisons using Fisher’s exact test revealed significant differences in gene prevalence between phylogenetic clusters (*p* < 0.05). Concordance analysis further demonstrated moderate-to-strong associations between plasmid replicons and ARG classes (Cramér’s V > 0.5) with IncF/IncHI plasmids showing strong coupling with *β*-lactamase genes (V = 0.58) and Col-type plasmids with *sul/dfrA* determinants (V = 0.51) indicating structured co-occurrence patterns rather than direct evidence of gene transfer. Despite localized clonal clustering, genetic diversity remained high (Simpson’s diversity index, D = 0.94), excluding single-clone expansion indicating a heterogeneous MDR population (Supplementary Table S2). No formal correction for multiple testing was applied; therefore, reported *p*-values should be interpreted with caution.

## 4 Discussion

The use of antimicrobials in poultry production particularly in low- and middle-income countries has elevated poultry farms as major reservoirs of antimicrobial-resistant bacteria (ARB) and ARGs (Wee et al., 2020; Kaesbohrer et al., 2019). Close proximity between animals, humans and shared environments in such rural poultry ecosystems facilitates continuous exchange of resistant microbes at the human-animal-environment interface reinforcing AMR as a critical One Health challenge (Aslam et al., 2021; Ahmad et al., 2023). However, in backyard poultry systems, antimicrobial exposure may be variable and not always well documented, and resistance may also arise through environmental exposure, contact with other livestock, or introduction of resistant strains from external sources. In this context, understanding the genetic characteristics, resistance mechanisms and transmission dynamics of *E. coli* circulating in backyard poultry settings is crucial to inform surveillance and mitigation strategies (Ghatak et al., 2021; Zhao et al., 2024).

In the present study, 45 out of 48 cloacal samples processed yielded *E. coli* reflecting its ubiquitous presence in avian gut microbiota. Phenotypic identification corroborated classical colony morphology and biochemical traits and mPCR using species-specific markers confirmed all isolates with high diagnostic confidence. This good culture recovery combined with molecular confirmation indicated the robustness of sampling and laboratory workflows and aligns with earlier Indian studies reporting high *E. coli* prevalence in poultry-rearing households (Hussain et al., 2019).

ESBL and *AmpC* genes were detected in 18 of 45 *E. coli* isolates, with *bla*_SHV_ (28.8%) and *bla*_TEM_ (26.6%) predominating, followed by *bla*_CTX-M_ (20%), a determinant widely associated with high-risk MDR clones; however, this should not be interpreted as direct evidence of strong *β*-lactam selective pressure within small-scale poultry production systems (Dierikx et al., 2020). The presence of resistance genes may also arise through potential genetic exchange mechanisms mediated by MGEs, independent of direct antibiotic exposure (Partridge et al., 2018; Gillings, 2014). The AmpC was detected in 11.11% of isolates including mixed ESBL-AmpC carriers indicating accelerated evolution of β-lactam resistance. These findings were consistent with the phenotypic double disc diffusion assay which revealed high resistance to multiple third-generation cephalosporin (cefpodoxime, ceftazidime, cefotaxime, ceftriaxone and aztreonam) reflecting typical ESBL/AmpC activity where these enzymes hydrolyze the β-lactam ring of antibiotics thereby inactivating their bactericidal function. This analogy was supported by statistical testing demonstrating high prevalence of ESBL/AmpC-producing *E. coli* compared to conservative baseline estimates (Z = 3.46, *p* < 0.001). These findings suggest that multiple factors, including environmental exposure and genetic acquisition mechanisms, may contribute to the observed resistance patterns rather than direct selection pressure alone. The complete susceptibility to colistin and high susceptibility to imipenem is consistent with their restricted use in veterinary settings. These antimicrobials should remain strictly restricted in livestock to preserve their critical importance in human medicine rather than implying their effectiveness in poultry systems (World Health Organization, 2019; World Organisation for Animal Health, 2021).

The resistome analysis further revealed a strong arsenal of chromosomal and plasmid-borne ARGs. Universal presence of intrinsic efflux systems (*acrABC, acrR, acrEF, emrAB, mdt* operons and *mar* regulon) and chromosomal β-lactamase genes (*ampC/ampH*) indicates multidrug-tolerant genomic backbone. Acquired ARGs including *bla*_CTX-M-15,_ *bla*_TEM_ variants, plasmid-derived AmpC (*bla*_DHA-1_), *qnrS/B, dfrA1/14/17, sul1/2/3, tet(A), mph* genes and *aadA5* indicate diverse resistance-associated features probably linked to genetic acquisition processes, driven by antimicrobial exposure (Johansson et al., 2021; McMillan et al., 2019). ARG burdens differed significantly between phylogroups (one-way ANOVA, *p* = 0.036) indicating lineage-specific acquisition dynamics. Further, moderate-to-strong concordance between plasmid replicons and ARG classes (Cramér’s V > 0.5) confirms that resistance architecture is non-random and primarily plasmid-driven. The occurrence of multiple ARGs/isolate underscores the polyresistant nature of poultry *E. coli* consistent with regional and global reports (Young et al., 2022; Zhao et al., 2024; Pourahmad and Jazayeri, 2013; Das et al., 2022).

Virulence gene profiling demonstrated a virulome comprising adhesion factors (*fim, csg* and *ec*p), iron acquisition systems (*ent, fep, fes* and *ybt* clusters), stress resistance determinants (*gad, hlyE, ompT, iss*) and secretion system genes (*gsp* operon) (Maluta et al., 2017; Ouellette et al., 2022). Such combinations suggest a mixture of commensal, APEC-like and ExPEC-like traits (Braz et al., 2020; Maluta et al., 2017). Iron acquisition operons, especially enterobactin and yersiniabactin clusters are known to enhance bacterial fitness in nutrient-limited environments such as the avian gut (Wei and Murphy, 2016). The widespread detection of curli and type 1 fimbriae indicates strong colonization capability and potential for biofilm development traits that support environmental persistence and AMR survival (Renzhammer et al., 2020; Yan et al., 2020; Ouellette et al., 2022). Fisher’s exact test demonstrated significant co-occurrence between major adhesion genes (*fimH*, *csgA*) and biofilm-associated loci (*ecpA*, *ompA*) (*p* < 0.01).

Our analysis revealed that the *E. coli* resistome and virulome are organized into distinct functional modules reflecting the organized genome dynamics characteristic of the species where specific operons and MGEs contribute to variation in genomic profiles (Touchon et al., 2009). The previously described spatial correlation between *bla*_CTX-M-15_ and virulence factors such as *nlpI* and *ybt* is more appropriately interpreted as co-occurrence within specific genomic backgrounds rather than spatial or mechanistic association (Biggel et al., 2022). These findings indicate that while *ampC* provides a baseline genetic scaffold, the clinical trajectory of an isolate is ultimately governed by the presence of *qnr, sul,* and *fae* clusters likely reflects acquisition of these features. This modular architecture allows for the stratification of isolates into high-risk categories based on their integrated resistance and biofilm-forming potential, facilitating targeted genomic surveillance.

The 34 biofilm-associated gene repertoire further reinforces these traits. Universal detection of the curli operon (*csgA–G*) and broad distribution of type 1 fimbriae (*fimA–I*) along with *luxS* (quorum sensing), motility genes (*mot, fli*) and global stress regulators (*rpoS, hfq*) suggest that the isolates possess genetic determinants associated with environmental persistence and colonization (Sharma et al., 2016; Niu et al., 2013). Strong biofilm-forming potential is a major driver of AMR persistence by shielding bacteria from environmental stressors and antimicrobial agents (Das et al., 2022; Klein et al., 2018; Laganenka et al., 2016; Pourciau et al., 2020).

Analysis of plasmid replicons revealed a mosaic plasmid architecture dominated by IncFII, IncFIB, IncHI1A/HI1B and multiple Col-type plasmids. The IncF and IncHI plasmids are known carriers of ESBL and PMQR genes and are frequently implicated in MDR dissemination in poultry and humans (Jarocki et al., 2020). The presence of up to 11 plasmids in some isolates together with MDR-associated replicons indicates complex plasmid profiles within individual isolates; however, this should be interpreted as co-occurrence rather than direct evidence of plasmid-mediated transfer between strains (Miranda et al., 2016).

The MGE analysis demonstrated extensive genomic mobility with MITEEc1 present in all isolates followed by IS609, ISEc1, ISEc5, IS102 and IS30. Notably, IS26, one of the most clinically important insertion sequences associated with structurally complex resistance regions was present in nearly half of the isolates and was observed in isolates also carrying ESBL, *tet(A), qnr, sul* and *dfrA* genes (Johansson et al., 2021). MGEs carrying virulence factors such as *csg, fimH, ompT, yghJ* and *terC* indicate association between mobility elements and virulence-associated features at the isolate level, rather than direct evidence of adaptive transfer mechanisms (Jacob et al., 2015; Ludden et al., 2018).

CRISPR-Cas profiling revealed that 83.3% of isolates carried Type I-E CRISPR systems with high-evidence-level arrays. While such coexistence has been reported in MDR *E. coli*, it should be interpreted cautiously as the present analysis does not include functional validation such as spacer–protospacer matching or anti-CRISPR detection (Tantoso et al., 2022). Therefore, these isolates may represent strains carrying both CRISPR elements and multiple resistance/virulence determinants, but their functional interaction remains to be determined. These isolates represent a subset of poultry *E. coli* with combined MDR and virulence-associated traits with potential public health implications.

MLST revealed considerable clonal diversity with 12 distinct sequence types. Two STs (ST48 and ST3107) were shared by multiple isolates, suggesting common genomic backgrounds or shared sources rather than definitive dissemination (Mukerji et al., 2023). Despite this genetic diversity was high (Simpson’s D = 0.94), excluding expansion of a single dominant clone indicating a heterogeneous MDR population shaped by repeated introductions and independent acquisition events. The predominance of commensal phylogroups A (83.3%) and B1 (16.7%) aligns with typical avian gut flora. The presence of ESBL/*AmpC* genes and virulence traits within these phylogroups placed the 18 NIVEDI isolates into two major clades within phylogroups A and B1, characterized by limited genomic divergence and a restricted repertoire of MLST sequence types. When compared with 105 global genomes from China, Brazil, Ghana, Cuba, Ecuador, India, Bangladesh, and Thailand, the Indian isolates clustered together suggesting genetic similarity within the dataset while retaining shared ancestry with globally distributed commensal lineages (Wee et al., 2020). The dominance of commensal STs and the absence of admixture with extraintestinal phylogroups B2, D, and F indicated limited representation of highly virulent lineages in poultry birds. Nevertheless, several of the identified STs were globally distributed and frequently associated with AMR reservoirs in food animals. Thus, despite their commensal phylogenetic placement, the MDR and virulence-associated traits observed in these isolates represent a possible zoonotic and food-chain risk (Braga et al., 2016), highlighting the role of poultry as a reservoir of resistant commensal *E. coli* rather than confirming active dissemination pathways.

Overall, the convergence of extensive ARGs, MGEs, multidrug efflux systems, high-risk plasmids, biofilm-forming genes and diverse virulence factors suggests that backyard poultry may act as reservoirs of MDR *E. coli* (Hickman et al., 2021). These isolates demonstrate the ability to persist and adapt within poultry environments; however, direct transmission or dissemination across animals, environment, and humans was not assessed in this study and should not be inferred from the present data (Zhao et al., 2024). Strengthening antimicrobial stewardship, improving biosecurity and implementing genomic AMR surveillance are rightly needed to mitigate the emergence and spread of MDR *E. coli* within poultry production chains.

However, this study is subject to certain limitations. For example, the enrichment of fecal samples may have biased the results towards faster-growing strains potentially under-representing the full diversity of *E. coli*. Second, the selection of a single isolate/sample/plate while standard for assessing population diversity, limits the capture of within-host strain heterogeneity. Third, WGS of a small subset of 18 isolates underscores the need for larger-scale surveys. Fourth, resistance, virulence, and biofilm genes were identified *in silico*, confirming genetic potential but not phenotypic expression. Additionally, the absence of pairwise SNP distance analysis limits the ability to infer recent transmission events. Collectively, these limitations suggest our results should be interpreted as exploratory and validated with larger and more representative datasets. All associations described in this study are based on isolate-level co-occurrence of genomic features and should not be interpreted as evidence of physical linkage, HGT or direct transmission. Overall, poultry reared at backyards in smallholder systems serve as important reservoirs of genetically versatile MDR *E. coli*, capable of transmitting resistance and virulence determinants across the animal–environment–human interface. These findings reinforce the need for enhanced AMR surveillance, responsible antimicrobial use and strengthened biosecurity within poultry production under a One Health framework.

## 5 Conclusion

This study provides an in-depth phenotypic and genomic overview of MDR *E. coli* circulating in poultry birds rared under backyard farming system in southern India. All isolates carried resistome with a high prevalence of ESBL genes (*bla*_SHV_, *bla*_TEM_, *bla*_CTX-M_) and plasmid-mediated AmpC, indicating the presence of *β*-lactam resistance determinants rather than direct evidence of selection pressure, supported by significant enrichment of resistant phenotypes (*p* < 0.001). The resistome further included diverse tetracycline, sulfonamide, quinolone and trimethoprim resistance genes. Virulome profiling revealed the presence of key adhesion, iron acquisition and stress tolerance genes while universal curli and type 1 fimbrial operons indicated high biofilm-forming potential, supporting environmental persistence. MGE including IS26, ISEc1, IS609 and MDR-associated IncF/IncHI plasmids were frequently observed in association with major ARGs; however, these patterns represent co-occurrence at the isolate level and do not provide direct evidence of horizontal gene transfer. CRISPR-Cas analysis revealed a high prevalence of Type I-E systems coexisting with multiple plasmids; however, the functional implications of this coexistence could not be determined in the absence of spacer–target matching or anti-CRISPR analysis and should be interpreted descriptively. MLST and serotyping demonstrated considerable genetic diversity (Simpson’s D = 0.94) with shared sequence types indicating common genomic backgrounds rather than definitive evidence of localized clonal spread or transmission. Core genome SNP-based phylogenomics showed that these isolates cluster within globally circulating commensal phylogroups A and B1, yet harbor MDR and virulence traits with zoonotic relevance. Overall statistical analysis indicates that backyard poultry-associated *E. coli* represent a reservoir of diverse MDR and virulence-associated determinants within the sampled population. While these isolates demonstrate the potential for persistence and adaptation, direct evidence of spread or zoonotic transmission was not assessed in this study. These findings provide baseline genomic insights to support antimicrobial resistance surveillance, responsible antimicrobial use, and improved biosecurity within poultry production systems under a One Health framework.

## Data Availability

The datasets presented in this study can be found in online repositories. The names of the repository/repositories and accession number(s) can be found in the article/Supplementary material.

## Footnotes

1
https://www.bioinformatics.babraham.ac.uk/projects/fastqc/

2
https://github.com/OpenGene/fastp

3
https://github.com/usadellab/Trimmomatic

4
https://github.com/tseemann/shovill

5
https://github.com/ablab/spades

6
https://github.com/combogenomics/medusa

7
https://github.com/ParBLiSS/FastANI

8
https://www.ncbi.nlm.nih.gov/genome/annotation_prok/

9
https://github.com/tseemann/prokka

10
https://cge.food.dtu.dk/services/ResFinder/

11
https://card.mcmaster.ca/analyze/rgi

12
https://cge.food.dtu.dk/services/VirulenceFinder/

13
https://cge.food.dtu.dk/services/PlasmidFinder/

14
https://cge.food.dtu.dk/services/MobileElementFinder/

15
https://crisprcas.i2bc.paris-saclay.fr/

16
https://cge.food.dtu.dk/services/MLST/

17
https://cge.food.dtu.dk/services/SerotypeFinder/

18
https://github.com/A-BN/ClermonTyping

19
https://cge.food.dtu.dk/services/CHTyper/

20
https://www.r-project.org/

21
http://tree.bio.ed.ac.uk/software/iqtree/

22
https://itol.embl.de/
