# Supplementary material for: Genomic profiling of ESBL/AmpC-producing Escherichia coli from backyard poultry: resistome, virulome, plasmidome, and CRISPR-Cas insights
Source: Front Microbiol. 2026 May 29;17:1836952. doi: 10.3389/fmicb.2026.1836952 (PMC13260132; doi:10.3389/fmicb.2026.1836952)
Supplement: Supplementary file 1 [file Table_1.DOCX]

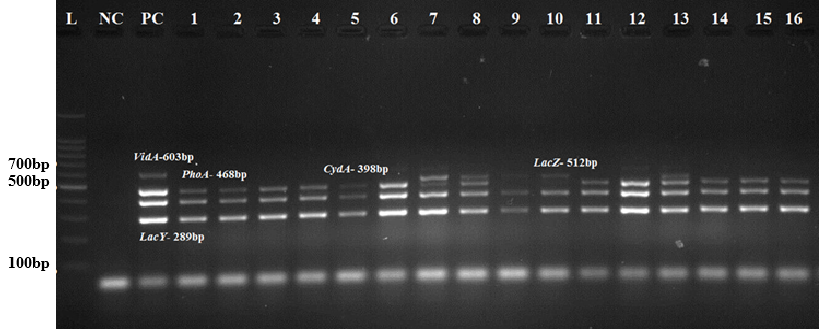


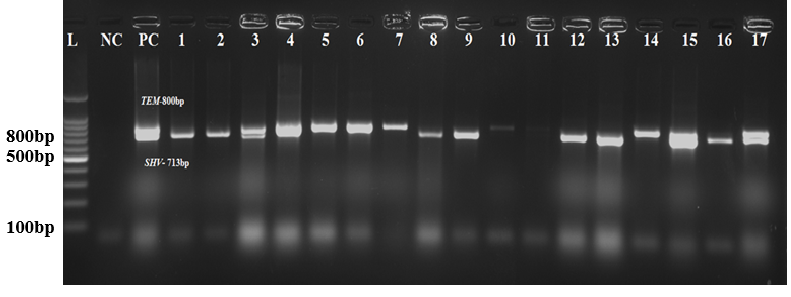
**Supplementary figure 1A.** Multiplex PCR-based detection of E. coli isolates. Agarose gel electrophoresis showing amplification of vidA (603 bp), phoA (468 bp), cydA (398 bp), lacY (289 bp), and lacZ (512 bp). Lane L: 100 bp DNA ladder; NC: negative control; PC: positive control (ATCC 25922); Lanes 1–16: representative E. coli isolates. Expected amplicon sizes are indicated alongside the bands.

**Supplementary figure 1B.** PCR-based detection of ESBL-associated genes in *E. coli* isolates. Agarose gel electrophoresis showing amplification of *blaTEM* (800 bp) and *blaSHV* (713 bp) genes. Lane L: 100 bp DNA ladder; NC: negative control; PC: positive control (ATCC 25922); Lanes 1–17: representative *E. coli* isolates. Expected amplicon sizes are indicated alongside the corresponding bands.


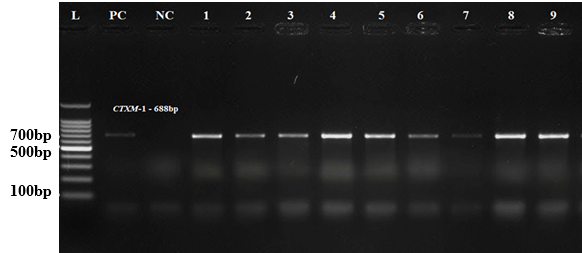


**Supplementary figure 1C.** PCR-based detection of the ESBL gene blaCTX-M-1 in E. coli isolates. Agarose gel electrophoresis showing amplification of the blaCTX-M-1 gene (688 bp). Lane L: 100 bp DNA ladder; PC: positive control; NC: negative control; Lanes 1–9: representative E. coli isolates (ATCC 25922). The expected amplicon size is indicated alongside the corresponding band.


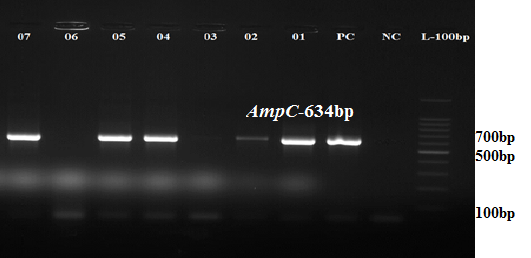


**Supplementary figure 1D.**  PCR-based detection of the AmpC β-lactamase gene in E. coli isolates. Agarose gel electrophoresis showing amplification of the AmpC gene (634 bp). Lanes 01–07: representative E. coli isolates; PC: positive control (ATCC 25922); NC: negative control; L-100 bp ladder. The expected amplicon size is indicated alongside the corresponding band.


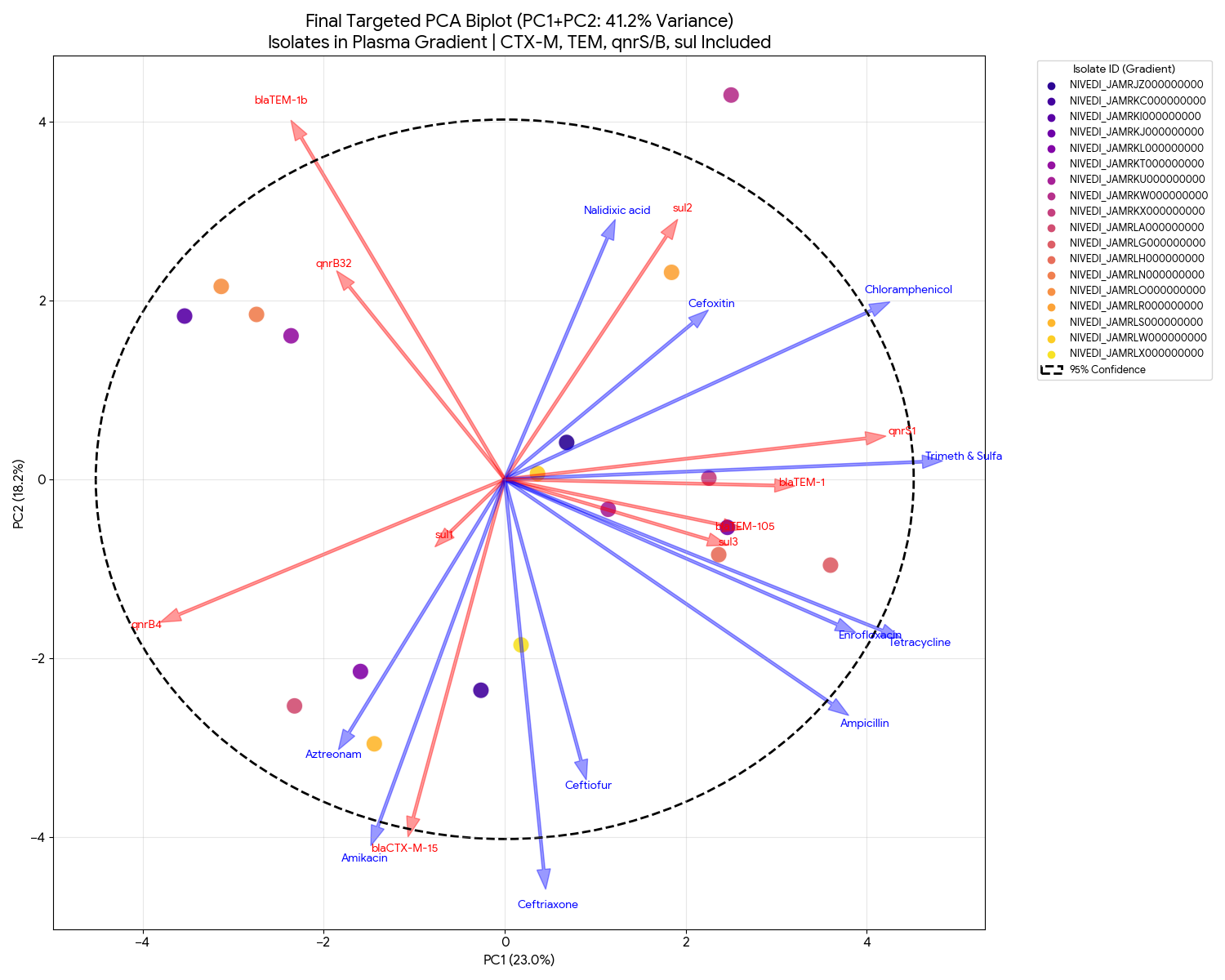


**Supplementary figure 2.** This biplot visualizes the relationship between the isolates and the overall resistance of ABST and ARGs
