# Supplementary material for: Genomic profiling of ESBL/AmpC-producing Escherichia coli from backyard poultry: resistome, virulome, plasmidome, and CRISPR-Cas insights
Source: Front Microbiol. 2026 May 29;17:1836952. doi: 10.3389/fmicb.2026.1836952 (PMC13260132; doi:10.3389/fmicb.2026.1836952)
Supplement: Supplementary file 2 [file Table_2.DOCX]

| **SL.NO** | **GenBank Accessions** | **Isolation country** | **Geographic group** | **Phylogroup** | **Sequence type** | **Isolation source** | **Host** |
| --- | --- | --- | --- | --- | --- | --- | --- |
| 1 | JANFDL000000000 | India | Asia | D | 69 | fecal | Poultry |
| 2 | JANFDK000000000 | India | Asia | D | 349 | fecal | Poultry |
| 3 | JANFDM000000000 | India | Asia | A | 656 | fecal | Poultry |
| 4 | JANFDJ000000000 | India | Asia | A | 484 | fecal | Poultry |
| 5 | JANFDI000000000 | India | Asia | B1 | 155 | fecal | Poultry |
| 6 | JANFGH000000000 | India | Asia | B1 | 155 | fecal | Poultry |
| 7 | JANFFP000000000 | India | Asia | A | **735** | fecal | Poultry |
| 8 | JALPOL000000000 | China | Asia | A | 871 | stool | Poultry |
| 9 | JANFEN000000000 | China | Asia | B1 | 101 | feces | Poultry |
| 10 | JANGRG000000000 | Ghana | Africa | A | 10 | cecal | Poultry |
| 11 | JANGRX000000000 | Ghana | Africa | A | 524 | cecal | Poultry |
| 12 | JANGQU000000000 | Ghana | Africa | D | 38 | cecal | Poultry |
| 13 | JANGRQ000000000 | Ghana | Africa | A | 710 | cecal | Poultry |
| 14 | JANGRL000000000 | Ghana | Africa | A | 206 | cecal | Poultry |
| 15 | JANGRT000000000 | Ghana | Africa | A | 1421 | cecal | Poultry |
| 16 | JANGRC000000000 | Ghana | Africa | A | 48 | cecal | Poultry |
| 17 | JANGRB000000000 | Ghana | Africa | B1 | 156 | cecal | Poultry |
| 18 | JANGRP000000000 | Ghana | Africa | A | 1722 | cecal | Poultry |
| 19 | CP073943 | Cuba | North America | A | 6876 | Cloacal | Poultry |
| 20 | CP073936 | Cuba | North America | A | 5334 | Cloacal | Poultry |
| 21 | CP073932 | Cuba | North America | A | 10 | Cloacal | Poultry |
| 22 | CP073924 | Cuba | North America | B1 | 156 | Cloacal | Poultry |
| 23 | CP073958 | Cuba | North America | A | 542 | Cloacal | Poultry |
| 24 | CP073946 | Cuba | North America | A | 656 | Cloacal | Poultry |
| 25 | CP073962 | Cuba | North America | A | 167 | Cloacal | Poultry |
| 26 | CP073953 | Cuba | North America | B1 | 953 | Cloacal | Poultry |
| 27 | CP073949 | Cuba | North America | A | 7101 | Cloacal | Poultry |
| 28 | CP073981 | Cuba | North America | A | 226 | Cloacal | Poultry |
| 29 | CP073974 | Cuba | North America | A | 6335 | Cloacal | Poultry |
| 30 | CP073976 | Cuba | North America | A | **1634** | Cloacal | Poultry |
| 31 | CP073988 | Cuba | North America | A | 1716 | Cloacal | Poultry |
| 32 | JAJALO000000000 | Brazil | South America | A | 93 | fecal | Poultry |
| 33 | JAJALZ000000000 | Brazil | South America | B1 | 224 | fecal | Poultry |
| 34 | JAJAMG000000000 | Brazil | South America | B1 | 224 | fecal | Poultry |
| 35 | JAJAMK000000000 | Brazil | South America | F | 10064 | fecal | Poultry |
| 36 | JAJAMR000000000 | Brazil | South America | F | 354 | fecal | Poultry |
| 37 | JAJANB000000000 | Brazil | South America | D | 2309 | fecal | Poultry |
| 38 | JAJANJ000000000 | Brazil | South America | B1 | 1158 | fecal | Poultry |
| 39 | JAJANN000000000 | Brazil | South America | A | 93 | fecal | Poultry |
| 40 | JAJANP000000000 | Brazil | South America | B1 | 2607 | fecal | Poultry |
| 41 | JAJANQ000000000 | Brazil | South America | D | 2309 | fecal | Poultry |
| 42 | JAJANR000000000 | Brazil | South America | B1 | 155 | fecal | Poultry |
| 43 | JAJANT000000000 | Brazil | South America | B1 | 2607 | fecal | Poultry |
| 44 | JAJANU000000000 | Brazil | South America | B1 | 224 | fecal | Poultry |
| 45 | JAJANV000000000 | Brazil | South America | B1 | 2607 | fecal | Poultry |
| 46 | JAJANW000000000 | Brazil | South America | B1 | 117 | fecal | Poultry |
| 47 | JAJANZ000000000 | Brazil | South America | B1 | 2607 | fecal | Poultry |
| 48 | JAJAOA000000000 | Brazil | South America | B1 | 2607 | fecal | Poultry |
| 49 | RXLF00000000 | Ecuador | South America | B1 | 602 | fecal | Poultry |
| 50 | RXLG00000000 | Ecuador | South America | F | 354 | fecal | Poultry |
| 51 | RXLE00000000 | Ecuador | South America | A | 191 | fecal | Poultry |
| 52 | RXLH00000000 | Ecuador | South America | B1 | 665 | fecal | Poultry |
| 53 | RXLJ00000000 | Ecuador | South America | B1 | 156 | fecal | Poultry |
| 54 | JBHJCU000000000 | Bangladesh | Asia | F | 648 | fecal | Poultry |
| 55 | JBHZLK000000000 | China | Asia | A | 7366 | fecal | Poultry |
| 56 | JBHZLZ000000000 | China | Asia | B1 | 2165 | fecal | Poultry |
| 57 | JBHZMI000000000 | China | Asia | A | 6782 | fecal | Poultry |
| 58 | JBHZMJ000000000 | China | Asia | D | 69 | fecal | Poultry |
| 59 | JBHZML000000000 | China | Asia | A | 48 | fecal | Poultry |
| 60 | JBHZMO000000000 | China | Asia | B1 | 1056 | fecal | Poultry |
| 61 | JBHZMQ000000000 | China | Asia | B1 | 3076 | fecal | Poultry |
| 62 | JBHZMT000000000 | China | Asia | A | 4015 | fecal | Poultry |
| 63 | JBHZMU000000000 | China | Asia | D | 2001 | fecal | Poultry |
| 64 | JBHZMS000000000 | China | Asia | A | 4429 | fecal | Poultry |
| 65 | JBHZMW000000000 | China | Asia | B1 | 156 | fecal | Poultry |
| 66 | JBHZMZ000000000 | China | Asia | A | 2973 | fecal | Poultry |
| 67 | JBHZMY000000000 | China | Asia | B1 | 162 | fecal | Poultry |
| 68 | JBHZMX000000000 | China | Asia | B1 | 156 | fecal | Poultry |
| 69 | JBHZNA000000000 | China | Asia | B1 | 224 | fecal | Poultry |
| 70 | JBHZNC000000000 | China | Asia | F | 117 | fecal | Poultry |
| 71 | JBHZNB000000000 | China | Asia | F | 117 | fecal | Poultry |
| 72 | JBHZND000000000 | China | Asia | A | 11956 | fecal | Poultry |
| 73 | JBHZNF000000000 | China | Asia | F | 117 | fecal | Poultry |
| 74 | JBHZNE000000000 | China | Asia | B2 | 174 | fecal | Poultry |
| 75 | JBHZNG000000000 | China | Asia | A | 2462 | fecal | Poultry |
| 76 | JBHZNI000000000 | China | Asia | A | **93** | fecal | Poultry |
| 77 | JBHZNH000000000 | China | Asia | F | 117 | fecal | Poultry |
| 78 | JBHZNJ000000000 | China | Asia | B1 | 224 | fecal | Poultry |
| 79 | JBHZNK000000000 | China | Asia | B1 | 162 | fecal | Poultry |
| 80 | JBHZNL000000000 | China | Asia | A | 1912 | fecal | Poultry |
| 81 | JBHZNM000000000 | China | Asia | B1 | 58 | fecal | Poultry |
| 82 | JBHZNN000000000 | China | Asia | A | 6756 | fecal | Poultry |
| 83 | JBHZNO000000000 | China | Asia | A | 117 | fecal | Poultry |
| 84 | JBHZNQ000000000 | China | Asia | A | 1638 | fecal | Poultry |
| 85 | JBHZNP000000000 | China | Asia | A | **9558** | fecal | Poultry |
| 86 | JBHZNR000000000 | China | Asia | F | 457 | fecal | Poultry |
| 87 | JBHZNT000000000 | China | Asia | F | 117 | fecal | Poultry |
| 88 | JBHZNV000000000 | China | Asia | B1 | 162 | fecal | Poultry |
| 89 | JBHZNU000000000 | China | Asia | B1 | 359 | fecal | Poultry |
| 90 | JBHZNX000000000 | China | Asia | D | 405 | fecal | Poultry |
| 91 | JBHZNW000000000 | China | Asia | B1 | 224 | fecal | Poultry |
| 92 | JBHZNZ000000000 | China | Asia | A | 167 | fecal | Poultry |
| 93 | JBHZNY000000000 | China | Asia | A | 617 | fecal | Poultry |
| 94 | JBHZOA000000000 | China | Asia | B1 | 156 | fecal | Poultry |
| 95 | JBHZOC000000000 | China | Asia | D | 349 | fecal | Poultry |
| 96 | JBHZOD000000000 | China | Asia | F | 117 | fecal | Poultry |
| 97 | JBHZOE000000000 | China | Asia | B2 | 95 | fecal | Poultry |
| 98 | JBHZOF000000000 | China | Asia | B1 | 224 | fecal | Poultry |
| 99 | JBHZOH000000000 | China | Asia | B1 | 156 | fecal | Poultry |
| 100 | JBHZOI000000000 | China | Asia | B1 | 5869 | fecal | Poultry |
| 101 | JBLUNT000000000 | Bangladesh | Asia | A | 6018 | fecal | Poultry |
| 102 | NIVEDI_JAMRLX000000000 | India | Asia | A | 216 | Cloacal | Poultry |
| 103 | NIVEDI_JAMRLW000000000 | India | Asia | B1 | **3576** | Cloacal | Poultry |
| 104 | NIVEDI_JAMRLN000000000 | India | Asia | A | 3107 | Cloacal | Poultry |
| 105 | NIVEDI_JAMRLO000000000 | India | Asia | A | 3107 | Cloacal | Poultry |
| 106 | NIVEDI_JAMRLH000000000 | India | Asia | A | 5834 | Cloacal | Poultry |
| 107 | NIVEDI_JAMRLG000000000 | India | Asia | A | **6856** | Cloacal | Poultry |
| 108 | NIVEDI_JAMRKX000000000 | India | Asia | A | 2936 | Cloacal | Poultry |
| 109 | NIVEDI_JAMRLA000000000 | India | Asia | B1 | **297** | Cloacal | Poultry |
| 110 | NIVEDI_JAMRKC000000000 | India | Asia | A | 226 | Cloacal | Poultry |
| 111 | NIVEDI_JAMRJZ000000000 | India | Asia | B1 | **3576** | Cloacal | Poultry |
| 112 | NIVEDI_JAMRLS000000000 | India | Asia | A | 48 | Cloacal | Poultry |
| 113 | NIVEDI_JAMRLR000000000 | India | Asia | A | 48 | Cloacal | Poultry |
| 114 | NIVEDI_JAMRKU000000000 | India | Asia | A | 2705 | Cloacal | Poultry |
| 115 | NIVEDI_JAMRKT000000000 | India | Asia | A | 10 | Cloacal | Poultry |
| 116 | NIVEDI_JAMRKW000000000 | India | Asia | A | 48 | Cloacal | Poultry |
| 117 | NIVEDI_JAMRKL000000000 | India | Asia | A | 165 | Cloacal | Poultry |
| 118 | NIVEDI_JAMRKJ000000000 | India | Asia | A | 206 | Cloacal | Poultry |
| 119 | NIVEDI_JAMRKI000000000 | India | Asia | A | 206 | Cloacal | Poultry |
| 120 | RKLH00000000 | Thailand | Asia | B1 | 101 | fecal | Poultry |
| 121 | RKLI00000000 | Thailand | Asia | A | 7986 | fecal | Poultry |
| 122 | RKLJ00000000 | Thailand | Asia | B1 | 101 | fecal | Poultry |
| 123 | JAENPM000000000 | India | Asia | B1 | 7361 | fecal | Calf |

**Supplementary Table S1.** Metadata of Escherichia coli genomes used for comparative phylogenomic analysis, including GenBank accession numbers, country of isolation, geographic region, phylogroup, sequence type (MLST), isolation source, and host (n = 123; 105 reference genomes and 18 NIVEDI poultry isolates).

| **Sl. No.** | **Result domain** | **Metric / comparison** | **Statistical test / measure** | **Outcome** | **Interpretation** |
| --- | --- | --- | --- | --- | --- |
| 1 | ESBL/AmpC enrichment | Observed prevalence vs. baseline | One-sample Z-test | Z = 3.46, *p* < 0.001 | Significant enrichment of β-lactam-resistant isolates. |
| 2 | Class-wise resistance | Resistance across antibiotic classes | Chi-square test | *p* < 0.001 | Resistance is non-uniform and dominated by β-lactams. |
| 3 | Multidrug resistance | MDR status | Proportion summary | 100% MDR | All isolates are multidrug resistant. |
| 4 | Resistance burden | Resistant antibiotics per isolate | Mean ± SD | 9.4 ± 2.1 | Uniformly high MDR burden across isolates. |
| 5 | Lineage effect | ARG load by phylogroup | One-way ANOVA | *p* = 0.036 | Lineage-specific accumulation of ARGs. |
| 6 | Clade differences | Gene prevalence across clusters | Fisher’s exact test | *p* < 0.05 | Significant clade-specific gene enrichment. |
| 7 | Plasmid structuring | ARG classes vs. replicons | Cramér’s V | V > 0.5 | Plasmid-driven resistome architecture. |
| 8 | ESBL–plasmid coupling | IncF/IncHI vs. β-lactamases | Cramér’s V | V = 0.58 | Strong association of ESBLs with MDR plasmids. |
| 9 | Folate-pathway resistance | Col plasmids vs. *sul/dfrA* | Cramér’s V | V = 0.51 | Col plasmids structure sulfonamide/trimethoprim resistance. |
| 10 | Population diversity | MLST profiles | Simpson’s index | D = 0.94 | High heterogeneity; no single-clone dominance. |

**Supplementary Table ST2**. Integrated statistical summary of phenotypic and genomic analyses of poultry-associated E. coli.

| **Component** | **Eigenvalue** | **Proportion of Variance** | **Cumulative Variance** |
| --- | --- | --- | --- |
| PC1 | 30.22 | 24.52% | 24.52% |
| PC2 | 25.60 | 20.77% | 45.29% |
| PC3 | 19.27 | 15.63% | 60.92% |
| PC4 | 11.63 | 9.43% | 70.36% |
| PC5 | 7.80 | 6.33% | 76.69% |

**Supplementary table 3.** PCA the eigenvalues and the proportion of variance captured by the primary components in virulence gens and biofilm genes
